# Phospholipid Membrane Interactions of Model Ac-WL-X-LL-OH Peptides Investigated by Solid-State Nuclear Magnetic Resonance

**DOI:** 10.3390/membranes14050105

**Published:** 2024-05-01

**Authors:** Nicolai Etwin Alsaker, Øyvind Halskau, Bengt Erik Haug, Nathalie Reuter, Willy Nerdal

**Affiliations:** 1 Department of Chemistry, University of Bergen, Allégaten 41, N-5007 Bergen, Norway; bengt-erik.haug@uib.no (B.E.H.); nathalie.reuter@uib.no (N.R.); willy.nerdal@uib.no (W.N.); 2 Department of Biological Sciences, University of Bergen, Thormøhlensgate 53A, N-5006 Bergen, Norway; oyvind.halskau@uib.no; 3 Computational Biology Unit, Department of Informatics, University of Bergen, Thormøhlensgate 55, N-5008 Bergen, Norway

**Keywords:** solid-state NMR, peptide–membrane interactions, phospholipid membranes

## Abstract

The role of aromatic amino acids in peripheral protein membrane binding has been reported to involve cation–π interactions with choline lipids. In this study, we have investigated the interactions of the model pentapeptide Ac-WL-X-LL-OH (where X = L, Y, F, or W) with the phospholipid membrane using solid-state NMR. The effect of guest residue X on the peptide-lipid interactome was complementary to the seminal report on the interfacial hydrophobicity scale by Wimley and White. We found that the phospholipids retained a lamellar phase in the presence of each of the peptides with an aromatic X residue, whereas the Leu peptide perturbed the bilayer to an extent where an additional isotropic phase was observed. The solid-state NMR ^13^C and ^31^P data provide additional information on the influence of these short peptides on the membrane that has not been previously reported. The magnitude of membrane perturbation was in the order of guest residue X = L > Y~F > W, which is consistent with the relative amino acid interfacial affinity reported by Wimley and White. Further work is, however, required to uncover the behavior of the peptide and localization in the membrane domain due to ambiguity of the ^13^C NMR data. We have launched efforts in this regard for the objective of better understanding the role of aromatic amino acids in peripheral membrane protein binding.

## 1. Introduction

Peripheral membrane proteins (PMPs) or amphitropic proteins have a plethora of functions in cells, including being involved in cell signaling and membrane trafficking [1,2,3], lipid processing [4,5], and cell membrane remodelling [6,7,8]. PMPs interact with lipid membranes in a reversible manner, and their temporary attachment to cell or organelle membranes can be a key step in accomplishing their function [4,9]. The residence times of PMPs on membranes range from milliseconds to a few seconds. Consequently, information on interfacial binding as well as potential protein conformational changes upon membrane association are challenging to obtain. The size of protein amino acid sequences adds an additional obstacle in examining such systems, in which case, shorter model peptides are useful. In their seminal work, Wimley and White [10] employed HPLC equilibrium dialysis to quantify the partitioning of membrane-active pentapeptides (Ac-WL-X-LL-OH) into the zwitterionic interface of POPC bilayers to derive their widely-used hydrophobicity scale for each of the 20 natural amino acids. The aromatic amino acids were ranked at the top of their hydrophobicity scale [10] and have the highest interfacial membrane affinity. It has recently been reported that the aromatic residues in several peripheral membrane proteins may function as more than hydrophobic anchors and play a more comprehensive role in the interfacial recognition mechanism. Specifically, tyrosine and tryptophane are able to engage in cation–π interactions with choline lipid headgroups, but phenylalanine is not [11,12,13]. Molecular simulations and prediction of free energy of transfer of aromatic amino acids in POPC bilayers have suggested that tryptophane and tyrosine might either insert below the average phosphate plane, just as the non-polar phenylalanine, or they might be localized in the choline region engaging in cation–π interactions.

To obtain a better understanding of the role of the aromatic amino acids in PMP–membrane binding, it would be useful to determine their depth of membrane insertion using model peptides. Indeed, there are several established techniques for determining the localization of peptides in the membrane domain, such as fluorescence quenching [14] and solid-state NMR (ssNMR) [15,16]. White and coworkers concluded from ssNMR experiments with indole, the side-chain of Trp, and three indole-analogues that Trp resides at the membrane interface [17]. However, to our knowledge, there are no published studies on the depth of insertion of the residues that are associated with membrane interactions of PMPs, particularly the aromatic amino acids. Atomic-level insight can be gained via ssNMR of lipid membranes without perturbing the membrane, and this is a useful technique for studying the effects of any embedded molecules [15,16,17,18,19,20,21]. The amount of foreign components is generally low compared to the lipid and is below the detection limit of ^13^C NMR for direct analysis. Still, magic angle spinning (MAS) ^13^C NMR can give detailed information about the effects of peptide introduction on the lipid backbone, i.e., from the perspective of the lipid for the POPC/Ac-WL-X-LL-OH system. It should be pointed out that in their original paper, Wimley and White observed the peptide directly [10]. Determining depth of membrane insertion of foreign components has commonly been performed by isotope enrichment [17] or addition of paramagnetic probes [19,22,23,24]. NMR experiments such as REDOR [25] and dipolar recoupling experiments [26,27] have been successful in characterizing the effect of foreign components on lipid membranes and do not require additional sample preparation. Regular 1D ^13^C MAS NMR has also been used for estimating the insertion depth of introduced foreign components in a phospholipid membrane [20,28,29] and was chosen for the herein investigated POPC/Ac-WL-X-LL-OH system due to its simplicity. Additionally, ^31^P NMR can afford insights into lipid macrostructure(s) and dynamics. The statistical distribution of headgroup ^31^P nuclei give rise to characteristic static ^31^P line-shapes depending on the lipid phases [15,16,30], which are useful for determining organization/packing. ^31^P ssNMR has been proven to be an effective tool to determine the effect of small peptides on phospholipid membranes [31,32]. For instance, Naito et al. showed that the peptide dynorphin increases the temperature sensitivity of phospholipid membranes [33].

In this work, we have investigated POPC bilayers and the pentapeptide sequence Ac-WL-X-LL-OH employing ssNMR, where X = Phe, Tyr, or Trp, which is relevant to the question of possible cation–π interactions with POPC. Leucine (Leu) was also included for reasons of comparison, as it lacks aromaticity, whilst exhibiting comparable hydrophobicity with the aromatic amino acids [10]. It should be noted that all peptides include an N-terminal Trp_1_ residue and are thus, in principle, capable of engaging in cation–π interactions.

The presence of orientation-dependent anisotropic interactions in ssNMR is a trade-off between resolution and information. Since the resonance frequency depends upon orientation in the external magnetic field, the ability of lipids to reorient in the membrane is reflected in chemical shift anisotropy (CSA). For phospholipids, J. Seelig defined ^31^P CSA as Δσ, taking into account headgroup axial symmetry along the bilayer normal due to rotational motion [34].

(1) $$∆\sigma =\sigma_{∥}-\sigma_{⊥}=\frac{3}{2}(\sigma_{∥}-\sigma_{i})$$

where σ_∥_ and σ_⊥_ are the shielding tensors along and perpendicular to the bilayer normal, respectively, and σ_i_ represents the isotropic chemical shift. Simply put, the ^31^P CSA of phospholipids is determined from the width of the static line-shape. The lipid bilayer morphology and fluidity are highly susceptible to changes in composition [35]. Upon the introduction of foreign components, lipids can be affected homogeneously, or distinct local environments can form [20,36]. This depends on the properties of the introduced species. The Ac-WL-X-LL-OH pentapeptide reportedly does not adopt a secondary structure and partitions exclusively into the membrane interface [37]. The spectral deconvolution of static ^31^P and MAS ^31^P NMR can reveal magnetically inequal phospholipid headgroups belonging to different environments.

## 2. Materials and Methods

### 2.1. Materials

Chemicals and solvents were purchased from Sigma-Aldrich (Schnelldorf, Germany) and used as received without any purification. 1-Palmitoyl-2-oleoyl-*sn*-glycero-3-phosphocholine (POPC) was purchased from Avanti Polar Lipids (Birmingham, AL, USA).

### 2.2. Peptide Synthesis

Full details for the synthesis and characterization of all the peptides are included in the Supporting Information. Briefly, all the peptides were synthesized using Fmoc-based solid-phase peptide synthesis on H-Leu-HMPB-ChemMatrix^®^ resin (Biotage, Uppsala, Sweden) using HCTU and DIPEA in DMF to facilitate amino acid coupling and 20% piperidine in DMF to facilitate Fmoc deprotection [38,39]. The N-terminus was acetylated using Ac_2_O/DIPEA/DMF (10:5:85, *v*/*v*) before cleavage from the resin using TFA/TIS/H_2_O (95:2.5:2.5 *v*/*v*). Precipitation in cold Et_2_O followed by purification on semi-preparative RP-HPLC gave the desired peptides with at least 95% purity (HPLC, 220 nm). The molecular composition was confirmed by HRMS, and the structure and amino acid sequence were confirmed by solution NMR.

### 2.3. Liposome Sample Preparation

POPC (100–200 mg scale, 0.132–0.254 mmol) suspended in CHCl_3_ (25 mg/mL) was mixed with Ac-WL-X-LL-OH peptide (10 mol%) in MeOH (~2 mL) and evaporated under a stream of argon, and then further under reduced pressure o.n. Deionized water that had been bubbled with Ar(g) was added to the dry sample to produce a suspension of approximately 20 mg lipid/mL. The suspension was homogenized by stirring at 40 °C for 6 h under an argon atmosphere, aided by manual stirring and sonication intermediately for the duration (any SUVs produced during sonication collapse with subsequent treatment [40]). Liposomes were prepared by subjecting the sample to at least 7 freeze–thaw cycles. Small amounts of NaOH (0.05–0.10 M) were added before each N_2_(l) treatment until reaching pH = 7.4, after which the suspension was lyophilized o.n. Fully hydrated MLV lipid samples [15] could then be obtained by the addition of an appropriate amount of argon-bubbled deionized water. The samples were equilibrated at 40 °C for 72 h under an argon atmosphere before packing in ssNMR rotors. The samples were mass controlled to have less than 4% variation. ^1^H MAS NMR confirmed the presence of peptides and 40–50 wt% water content for all the samples (spectra are included in the SI, Figures S14–S17). Dvinskikh et al. [41] noted a plateauing of the lipid dynamics above 40 wt% hydration for DMPC (full hydration), which is assumed to be similar for POPC.

### 2.4. Solid-State NMR

Solid-state NMR experiments were conducted on a wide-bore Bruker Ascend 500 MHz spectrometer (Fällanden, Switzerland) equipped with a PH MASDVT 500WB BL4 probe with a ^1^H/^31^P/^23^Na-^29^Si triple-resonance RF insert (Fällanden, Switzerland). The recording of the experiments were carried out with Bruker TopSpin^®^ 3.6.3. software. Pulse calibration was performed on adamantane spinning at 10 kHz for ^1^H and ^13^C following the TopSolids^tm^ workflow and on the sample of interest for ^31^P. Adamantane spinning at 10 kHz was used as the external references for ^1^H (1.85 ppm) and ^13^C (38.48 ppm) and static H_3_PO_4_ (85%) for ^31^P (0 ppm). The samples were packed into Kel-F MAS rotor inserts sealed with plugs and screws and placed inside 4 mm ZrO_2_ rotors with a Kel-F drive cap. ^1^H spectra were recorded with a 90^o^ pulse (113.6 kHz) for 1 transient. ^13^C spectra were recorded with a 90^o^ pulse (96.2 kHz) for 14k transients, 5 s recycle delay and ^1^H BB decoupling (spinal64, 35.7 kHz) during acquisition.^31^P static and MAS spectra were recorded with 16k and 512 transients, respectively, with a total of 1 s delay using 30^o^ pulses (46.3 kHz) and ^1^H BB decoupling (as above) during acquisition, unless otherwise stated. NMR experiments were performed at 298 K sample temperature, unless otherwise stated, and this was kept stable at ± 0.1 K using a Bruker BCU II—80/60 variable temperature unit (Fällanden, Switzerland). All MAS spectra were recorded with sample spinning rate of 6 kHz unless otherwise stated. The spectra were processed using TopSpin^®^ 4.1.1. ^13^C, and static ^31^P spectra were processed with line broadening of 5 Hz and 20 Hz, respectively. ^31^P NMR spectral deconvolution and carried out with the solid line-shape analysis (“sola”) software extension, which was also used to extract CSA. All the CSA values were adjusted by a factor 3/2 to be consistent with J. Seelig’s definition of Δσ [34]. All the simulated fits were generated to have >90% overlap with the experimental spectra, with δ_iso_ set to the chemical shift of the corresponding ^31^P MAS peak.

## 3. Results and Discussion

The four model pentapeptides Ac-WL-X-LL-OH containing Leu, Tyr, Phe, or Trp were prepared using Fmoc-based solid-phase peptide synthesis. Thereafter, the POPC liposome samples were prepared with each peptide present using a freeze–thaw protocol at a lipid/peptide ratio of 10:1, meanwhile adjusting the pH to 7.4. All the samples were hydrated to 40–50 wt% water content and equilibrated before analysis by ssNMR. The results are presented and discussed in this chapter.

### 3.1. ^31^P NMR

**Figure 1:**
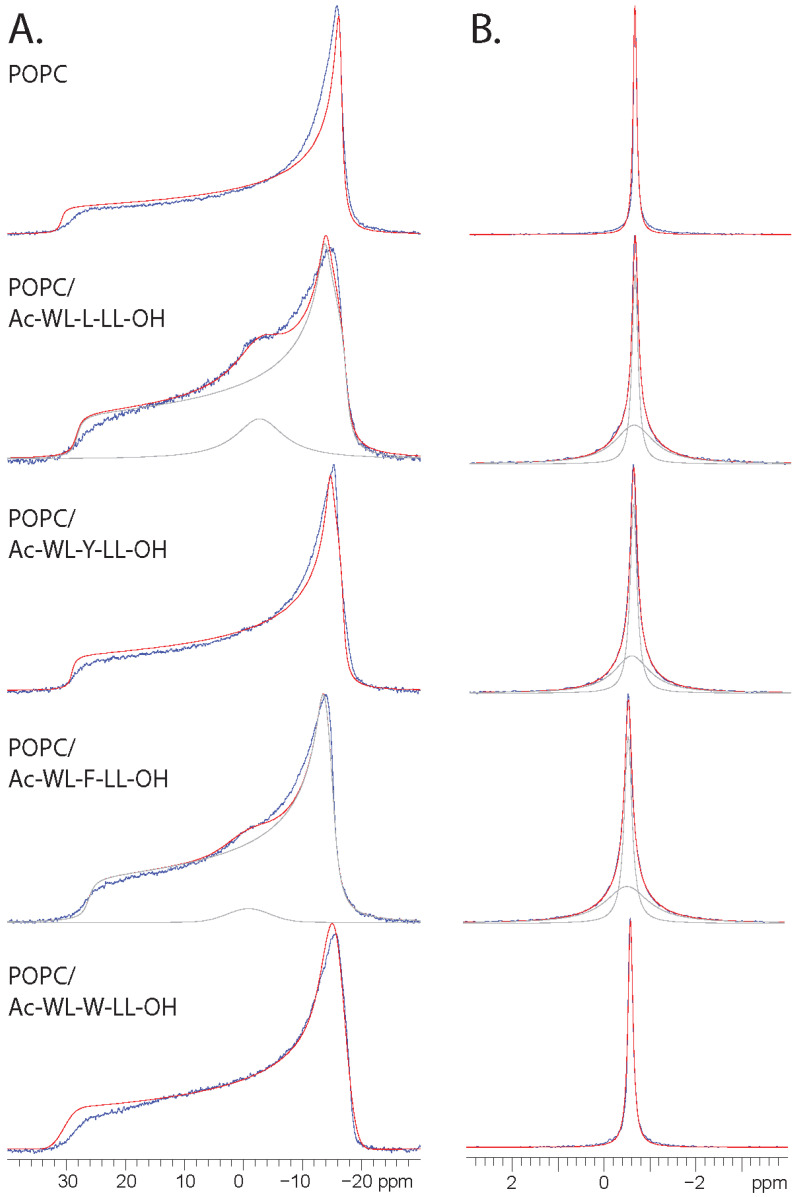
(**A**) The static ^31^P NMR spectra (blue) of POPC lipid samples doped with AcWL-X-LL-OH (10 mol%) at 298 K and approximately 50% hydration. The reconstructed spectra (red) are shown with the deconvolution of individual peaks where relevant (gray). The extracted CSA Δσ of the main lamellar line-shape signal in descending order in the figure legend: 47.3 ppm, 43.4 ppm, 44.7 ppm, 40.5 ppm, and 46.7 ppm. Isotropic component accounts for 13% and 3% of the lipids for the Leu and Phe peptides, respectively (**B**) Corresponding ^31^P MAS NMR experimental (blue) and reconstructed spectra (red), with deconvolution where relevant (gray). The extracted LB parameter of the lamellar POPC signal in descending order in the figure legend: 18 Hz, 38 Hz, 42 Hz, 46 Hz, and 26 Hz.

The static and MAS ^31^P NMR spectra of the POPC/Ac-WL-X-LL-OH samples are shown in Figure 1. The static ^31^P line-shape of pure POPC was expectedly consistent with a single liquid-crystalline lamellar phase [15,16]. This lamellar line-shape persisted in the static ^31^P spectra of all samples (see Figure 1A) for the majority of lipids, with CSA relative to POPC in the order of X = F < L < Y < W. Limited perturbation of the membrane was observed in presence of the Tyr and Trp peptides judging by small changes in the static ^31^P spectra compared to pure POPC. Only the ^31^P MAS spectrum (see Figure 1B) of the latter peptide sample is comparable to that of pure POPC (see Figure 1B), whereas this is not the case for the other three peptides (see discussion below).

A more profound influence of the Leu and Phe peptides on membrane morphology is revealed through the deconvolution of the static ^31^P spectra. An isotropic phase accounts for 13% and 3% of the lipids in the respective samples, where the line-shape was reminiscent of a micellar or cubic phase [15,42]. Cause of such isotropic signals are open for interpretation however, and can be attributed to different explanations [43]; foreign component inducing phase changes, intermediate motion vesicles [44] or different lipid headgroups motion (e.g., lipid in different topologies of the membrane). Regardless, in presence of the Leu peptide, a portion of the lipids evidently adopted a high mobility phase and abandoned bulk lamellar packing. The small amount of lipids in an isotropic phase in the Phe peptide sample can arguably be attributed to sample preparation. However, considering the influence on membrane dynamics, as indicated by CSA reduction, in comparison to POPC, the effect likely stems from the presence of the peptides. It is possible that the exchange between the two lamellar lipid environments is slower than the timescale of NMR at an ambient temperature and could belong to the same lipid phase. The corresponding ^31^P MAS spectra (see Figure 1B) indicated that this is likely true as only one isotropic shift was observed. In presence of the Leu-, Phe-, and Tyr peptides, the lipid ^31^P MAS peak FWHH (full width at half height) was approximately double that of the pure POPC reference sample. The deconvolution of the MAS spectra indicates the presence of two distinguishable phosphate sites associated with different line broadening, and by extension, lipid dynamics. The relative integrals do not coincide with the isotropic portion in the static reconstructions, thus indicating that the phospholipids in the lamellar organization have different mobility. This is known to occur in liposomes in high-curvature lamellar regions or for intermediate size vesicles [33]. Regardless of the reason, the ^31^P MAS spectra indicate increased heterogeneity of the POPC bilayer in the presence of Ac-WL-X-LL-OH for X = L, F, and Y.

In summary, these results indicate that the guest residue X is important for the peptide influence upon membrane dynamics. The Phe peptide induced the largest reduction in bilayer CSA (translating to an increase in lipid mobility), whereas the Leu peptide had the largest disruptive effect on the lipid macro structure, as revealed by spectral deconvolution (spectral reconstruction gave only satisfactory results for asymmetry > 0.1). The Trp peptide was indicated to have the smallest influence on the membrane and was comparable to the POPC reference sample. The peptides are ranked as follows based on influence on the membrane, X = L > F > Y > W, which is consistent with Wimley and White’s reported interfacial hydrophobicity scale [10]. An emphasis should be put on *interfacial*, as the ^13^C NMR experiments (see Section 3.3) revealed that the peptides perturbed at and/or below the membrane interface, suggesting that the peptides interact with the interface while embedded deeper. 

Wimley and White reported that unlike the other host/guest peptides, the Leu peptide displayed unusual properties [10]. While investigating the homologous hexapeptide Ac-WL_5_-OH, they found that it bound to liposomal POPC exponentially stronger at higher peptide/lipid concentrations (up to 3 mol%) due to cooperative binding, but this was reportedly not the case for Ac-WLLLL-OH [45]. Our ^31^P NMR data suggest that the same process of partitioning may occur at a higher peptide/lipid concentration (here 10 mol%) for Ac-WLLLL-OH. To dispel suspicions that this behavior could be an effect caused by the charged C-terminal carboxylate, we also investigated the amide analogue Ac-WLLLL-NH_2_ under the same conditions. For the POPC/Leu peptide amide sample, only the lamellar phase line-shape was present in the static ^31^P spectrum at 298 K (Figure S26 in SI). One lipid environment was confirmed by spectral deconvolution, indicating that the uncharged peptide did not perturb lamellar lipid packing. This suggests that the C-terminus is important for cooperative partitioning and aggregating behavior in the membrane. Interestingly, White et al. also reported that the neutral Ac-WLLLL-OMe did form β-sheets [45]. It appears that there are some inconsistencies regarding the membrane behavior of these Leu peptides between our and Wimley and White’s finding, although these are different systems. This investigation could be an interesting research direction. Due to these unique properties, it is likely that Leu is not an adequate hydrophobic control residue for comparison with the aromatic residues.

**Figure 2:**
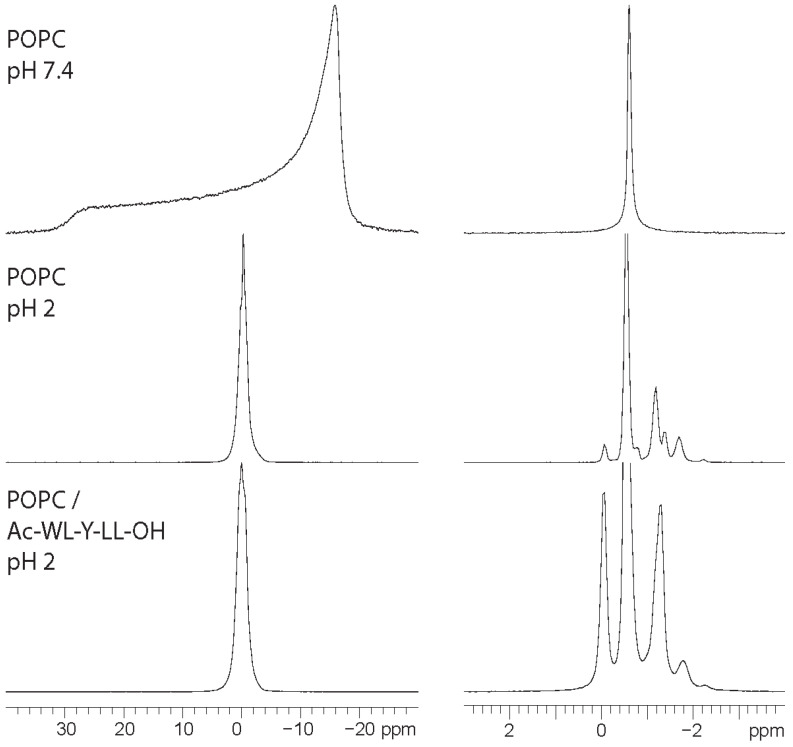
^31^P static (**left**) and MAS (**right**) spectra of hydrated POPC at pH 7.4 and 2, as well as for POPC doped with Ac-WL-Y-LL-OH (10 mol%).

At the investigated pH 7.4, the C-terminus is deprotonated and carries a negative charge, serving as an anchor point for the aqueous region and membrane interface. In their original paper, Wimley and White [10] also investigated the partitioning of Ac-WL-X-LL-OH at pH 2 for certain X amino acids. In our endeavor to uncover the role of the C-terminus in binding, we investigated these conditions, at which the peptide is neutral. However, at pH 2, the ^31^P NMR of pure POPC and of the peptide/lipid sample showed no similarity to a lamellar phase (Figure 2). At what pH this decomposition occurs is unsure. The pH interval 5.3–7.4 has been studied thoroughly with ssNMR, where substantial changes to the POPC membranes were reported already at a pH of 5.3 [46]. It should be noted that the bilayer remained intact at pH 5.3, as indicated by static and ^31^P MAS. To the best of our knowledge, this is the first time this has been reported and may have implications for the previously reported data at pH 2. We note that composition of lipid organization changed over the 1–2 weeks before reaching equilibrium, as indicated by a changes in the ^31^P MAS spectrum. 

### 3.2. ^31^P NMR Variable Temperature Study

**Figure 3:**
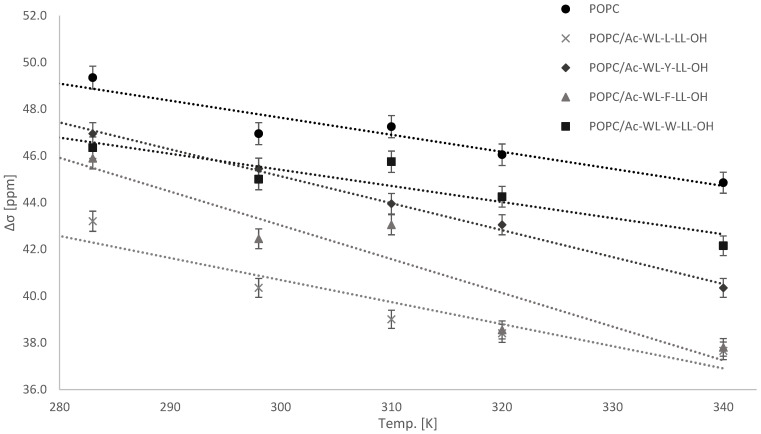
Static ^31^P line-shape extracted chemical shift anisotropy Δσ of the bulk lamellar phase for hydrated POPC lipid samples doped with peptide Ac-WL-X-LL-OH (10 mol%) at select temperatures. Errors were estimated based on the three simulation models in TopSpin^®^ solid line-shape analysis extension.

All the POPC/peptide samples were investigated at temperatures between 283 and 340 K and indicated the persistence of the guest residue rank order established in Section 3.1. The temperature range is above the POPC T_m_ of −2 °C; thus, the bilayer is fluid at all the investigated temperatures. The extracted Δσ of the static ^31^P NMR spectra (included in SI, Figures S19–S23) are plotted in Figure 3. A decrease in phospholipid CSA in the presence of Ac-WL-X-LL-OH was observed for all the guest residues X at all the investigated temperatures. The influence on CSA based on X followed the same order, L > F > Y > W, established at 298 K for all but one datapoint (which was within the estimated uncertainty). As expected, there were correlations between the bulk lamellar line-shape Δσ and temperature for all the samples, as is indicative of increased molecular motion in the phospholipid bilayer [33,43]. The temperature dependency of all the lipid/peptide systems followed a linear trend, with steeper curves than those for pure POPC, indicating the increased temperature sensitivity of phospholipid mobility in presence of the pentapeptides, with the exception of the Trp-peptide. 

The spectral simulation deconvolution of the static ^31^P spectra of the Leu peptide showed that the portion of lipids in the isotropic phase increased along with the temperature (see Figure S20 in SI). An explanation as to why the Leu peptide behaved significantly different to the others is again likely related to the possibility for cooperative partitioning, as discussed above. Compared to the other Leu peptide system, limited membrane perturbation was observed in presence of the other three peptides. However, at temperatures of 310 K and above, the effect of the Tyr and Phe peptides on POPC became apparent in the static ^31^P spectra (see Figures S21 and S22 in SI). Above these temperatures, the static ^31^P line-shape of both the peptide samples, of which Phe was most pronounced, became dissimilar to that of pure POPC at the elevated temperatures, which retained a lamellar phase. This indicates that the peptides have a substantial effect on membrane dynamics at elevated temperatures. A more-dampened effect was observed for the Trp peptide, again indicating that this peptide has the smallest influence on the lipid membrane. The well-known interfacial affinity of Trp [17,47,48] could offer an explanation, as localization at the interface would perturb the membrane to a lesser extent. 

### 3.3. ^13^C MAS NMR

The MAS ^13^C NMR analysis of all the POPC/Ac-WL-X-LL-OH samples was carried out and compared to that of a pure POPC reference sample. To determine the depth of insertion, several attempts were made at quantifying the effect of the peptides on specific ^13^C signals in the lipids upon peptide association to the membrane, relative to the pure POPC reference sample. The technique employed by Totland et al. [20] was unsuccessful in quantifying our system. Overly minor changes in the chemical shift of ^13^C peaks were observed (<0.06 ppm) for any meaningful trend to coalesce. We also attempted to quantify the effects of the peptides on the membrane via relative lipid ^13^C peak integral or signal intensity. However, this was not successful due to the poor S/N ratio for certain signals due to signal broadening. However, for relative intensity, the magnitude of the difference between the peptide/lipid samples and the reference POPC sample followed the same guest residue order as previously determined. Trends could be seen when quantifying the effect of peptide insertion based on the peak half-width (see Figure S26 in the SI). However, this too carries a level of uncertainty. Even slight changes in the chemical shift dramatically alter the shape of peaks constituted by more than one carbon signal (i.e., all lipid tail signals including the carbonyls), and consequently, the baseline for determining FWHH. Furthermore, the saturated carbon atoms between 3/3’ and 16/14’ could not be distinguished due to overlapping signals and poor resolution between 30 and 32 ppm. In brief, the ^13^C peak FWHH provides only qualitative information about membrane perturbation and was unsatisfactory as a quantitative measure of the investigated system. This holds especially true for the lipid tail region. Further work is required to accurately determine the depth of membrane insertion of the Ac-WL-X-LL-OH peptides, and as such, we have launched efforts to explore the 2D NMR space. Our results also indicate that the three aromatics behave differently, confirming earlier computational investigations that Tyr and Trp might engage in interactions at the level of the choline head groups. However, all the peptides investigated herein contain Trp, possibly constraining the peptides at the upper level of the interface.

**Figure 4:**
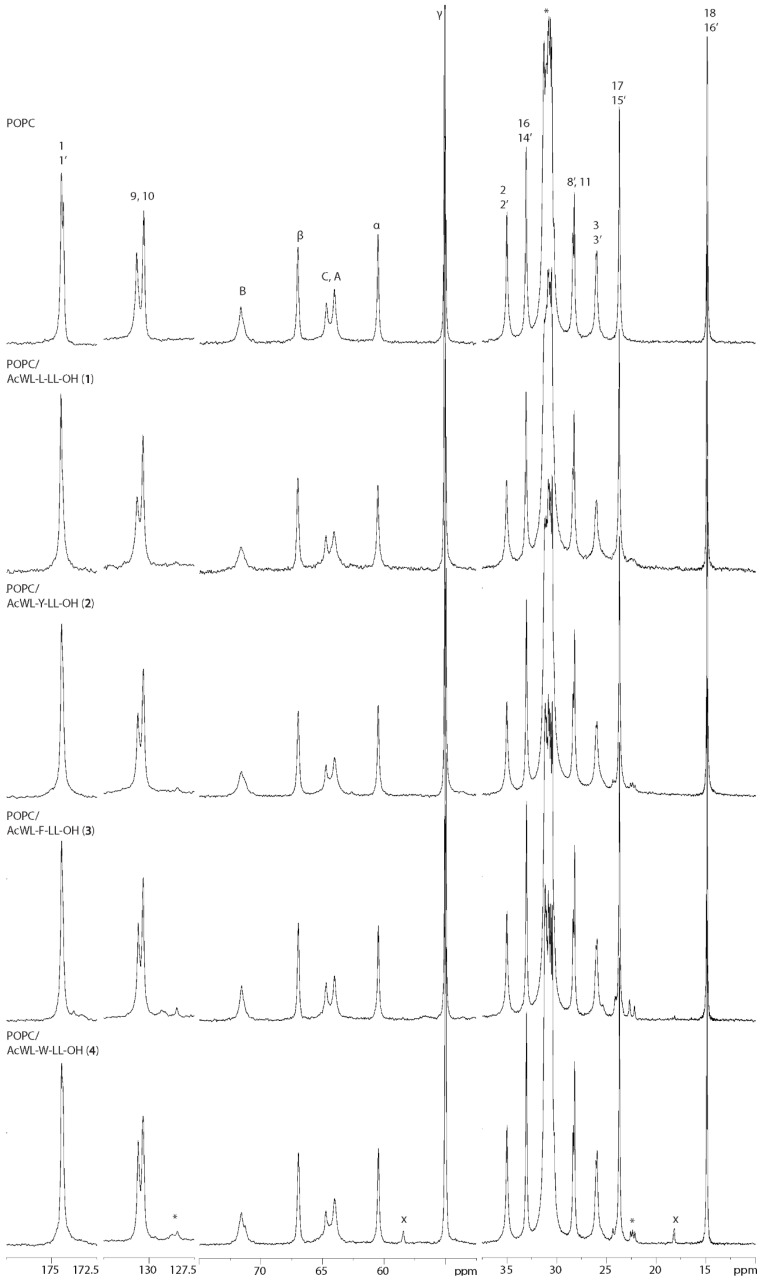
^13^C MAS NMR spectrum of hydrated POPC lipid samples doped with the peptide Ac-WL-X-LL-OH (10 mol%) for X = L, W. The influence of the Leu peptide upon insertion into the membrane is apparent from distortion (changes in shift, intensity, and width) of the glycerol and olefin 9 and 10 signals, especially in contrast to the similarities of pure POPC and POPC/Ac-WL-W-LL-OH. Residual peptide signals are marked with an asterisk (*). In the Trp peptide sample residual ethanol signals from cleaning the rotor (not in contact with sample) are marked with an x [51].

Despite the effect of peptides upon the lipid membrane not being quantifiable using 1D ^13^C MAS NMR, changes in certain lipid carbon signals are still apparent visually, which offers a qualitative assessment. The largest signal distortion with respect peak appearance relative to pure POPC was observed for the Leu peptide sample, specifically at the carbonyl 1/1’ and subsequent CH_2_ 2/2’ and 3/3’, the olefinic 9/10 and the glycerol moiety A, B, and C (see Figure 4). This is to be expected as these carbons are associated with relatively high order parameters and are unable to reorient in the membrane to a lesser extent than, e.g., the choline part or the CH_3_ termini (consistent with the previous observations [20]). The smallest effect upon the lipid carbon signals was for the Trp peptide, which was comparable to that of the reference POPC sample. Ranking the effect of the investigated qualitatively based on the magnitude of carbon signal distortion and the presence of residual peptide signals (see discussion below) for the various guest residues X gives an order consistent with that established from the ^31^P NMR data: L > Y ~ F > W. At first glance, the rank order appears to be inverse to Wimley and White’s scale [10]. However, the perspective of the analytical techniques must be considered. As ssNMR observes the lipid and the ^13^C and ^31^P data indicated that the Leu peptide is embedded deeper in the membrane than Trp peptide, i.e., the latter displays higher interfacial affinity. Ergo, the rank order is consistent with the amino acid interfacial hydrophobicity scale of Wimley and White [10]. Another likely possibility is that the peptide does not have a fixed location and behaves dynamically within the membrane matrix. This would explain the homogenous effect of the peptides on the membrane for both ^13^C and ^31^P NMR data (Leu peptide sample exception due to possible unique properties). Polarization transfer methods, which are selective for carbon mobility, such as a DP-CP-rINEPT series, have proven useful in assessing relative lipid dynamics [49,50]. In addition to segmentally characterizing carbon motion in POPC, it would be possible to determine the mobility of the peptide itself. However, this would require proportionally more experimental time without isotopic labelling at the present peptide/lipid ratio, and is dependent on relative mobility of the peptides. We note that residual peptide signals were not visible in our initial CP experiments (carried out for POPC/Ac-WL-F-LL-OH sample), as would be an expected observation for peptides which fluctuate within the membrane domain. This hypothesis is subject to further investigations.

**Figure 5:**
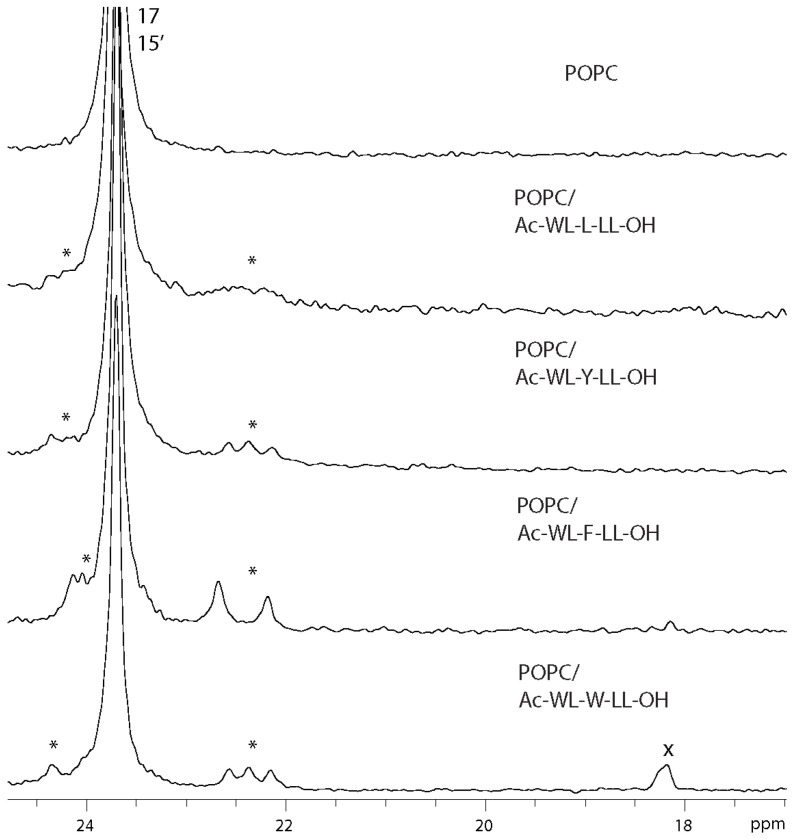
^13^C MAS NMR spectra of hydrated POPC lipid samples doped with peptides Ac-WL-X-LL-OH (10 mol%). Methyl CH_3_ signals from peptide Leu_2,4,5_ residues are marked with an asterisk (*). In the Trp peptide sample, the residual ethanol signals from cleaning the rotor (not in contact with sample) are marked with an x [51].

At 10 mol% peptide concentration, certain peptide carbon signals could be resolved, specifically Leu residue aliphatic carbons around 22–25 ppm and aromatic carbons at 128–129 ppm (see Figure 4 and Figure 5). The intensity and resolution of these signals were comparable for the aromatic peptides, whereas they were almost indiscernible from noise in the spectrum of POPC/Ac-WL-L-LL-OH. This decreased resolution indicates that the peptide has decreased dynamics, resulting in increased anisotropy on a timescale larger than what is averaged by the MAS, or reintroduced dipolar couplings from restricted motion. The same effect could be observed in the ^1^H MAS spectra for the residual peptide Ar-H and N-H signals between 6 and 8 ppm and the shoulder at ~0.8 ppm from the Leu_2,4,5_ CH_3_ signals (see Figures S14–S18 in SI). However, it is also possible that there are diffusion processes ongoing in the lipid matrix on a timescale larger than the NMR experiment and that line broadening stems from the changing local environment. Factors such as conformation, localization, insertion depth, and distribution contribute to the characteristics of broad peptide ^13^C signals. An alternative explanation could stem from reduced dynamics through the possibility of secondary β-sheet formation of the Leu peptide in the membrane domain (as discussed above), which would restrict its mobility. At present, this is only speculative and requires further investigations, but for the purpose of this study, we again note that X = L likely is not an adequate hydrophobic control residue for comparison to the aromatic residues. 

The effect of peptide on the membrane appeared independent of the peptide/lipid ratio within the concentration range tested, as the same results were apparent in the samples with 2.5, 5, and 10 mol% peptide added (analysis carried out for the POPC/Ac-WL-Y-LL-OH system). Separate lipid ^13^C signals originating from lamellar/isotropic lipids were expected for the Leu peptide sample. However, this was not observed at the investigated lipid/peptide ratio. That the phospholipids are organized in vesicles undergoing intermediate motion, motional frequencies which are not effectively averaged out by MAS [44], would offer an explanation, as it do the ^31^P data. The same is also suggested from our concurrent investigations into 2D ssNMR experiments. 

## 4. Conclusions

Solid-state NMR was used to provide atomic-level details about the interactions between POPC bilayers and four Ac-WL-X-LL-OH (X = L, Y, F, or W) model peptides. All the POPC membrane samples retained a lamellar phase in presence of the investigated peptides, where effect of the peptides upon lipid mobility occurred in order of the guest residues, X = L > Y ~ F > W, as indicated by the static ^31^P NMR CSA of the lamellar line-shape signal. Additionally, in presence of the Leu peptide, a portion of lipids adopted an isotropic phase. The Leu peptide and close relatives have been reported to exhibit cooperative partitioning and the ability to form secondary structures within the membrane domain [45], which offers an explanation for our observations. The rank order was supported by the magnitude of lipid signal distortion and residual peptide signals from ^13^C MAS NMR; however, the results are unambiguous due to the shortcomings of the 1D method for this system. Regardless, our ^31^P and ^13^C experiments provide additional information on the influence of these short peptides upon the membrane dynamics that have not been previously reported, complimentary with the relative amino acid interfacial affinity reported by Wimley and White [10]. In order to determine if the investigated short peptides fluctuate within the membrane domain, or if localization can be accurately pin-pointed, further work is required. Efforts are underway to shed light on this issue towards understanding at which level of the interface aromatic amino acids partition and the role of cation–π interactions in PMP–membrane binding.

## Data Availability

The original contributions presented in the study are included in the article/Supplementary Material, further inquiries can be directed to the corresponding author/s.

## Supplementary Materials

The following supporting information can be downloaded at: https://www.mdpi.com/article/10.3390/membranes14050105/s1. Additional experimental details, spectral data with assignment of synthesized peptides and ssNMR spectra are included in the Supporting Information (PDF). Tables S1–S4: Ac-WL-X-LL-OH ^1^H resonance tables for X = L, Y, F, W. Figures S1, S4, S8 and S11: ^1^H NMR spectrum of Ac-WL-X-LL-OH for X = L, Y, F, W. Figures S2, S6, S9, S12: HRMS ESI spectrum of Ac-WL-X-LL-OH for X = L, Y, F, W. Figures S3, S7, S10 and S13: Chromatogram (220 nm) of Ac-WL-X-LL-OH for X = L, Y, F, W. Figures S5: TOCSY spectrum of the NH region in F2 shows aromatic H^β^ that were suppressed during 1D ^1^HNMR with water suppression of Ac-WL-Y-LL-OH in CD_3_CN. Figures S14–S18: ^1^H NMR MAS spectrum of hydrated POPC sample doped with Ac-WL-X-LL-OH (10 mol%) for X = L, Y, F, W. Residual peptide signals (Ar-H and NH) are shown in the 7–9 ppm window. Figures S19–S23: Static ^31^P NMR signal line-shape of hydrated POPC sample doped with Ac-WL-X-LL-OH (10 mol%) for X = L, Y, F, W at select temperatures without BB decoupling. Figure S24: POPC carbon labelling. Figure S25: Peak width at half-height of specific ^13^C MAS signals originating from hydrated POPC samples doped with Ac-WL-X-LL-OH (10 mol%) relative to pure POPC. Figure S26: Static ^31^P NMR signal line-shape of hydrated POPC sample doped with Ac-WL-L-LL-NH_2_ (10 mol%) at 298 K without BB decoupling. Reference [52] is cited in Supporting Information.
