# Supplementary material for: Phospholipid Membrane Interactions of Model Ac-WL-X-LL-OH Peptides Investigated by Solid-State Nuclear Magnetic Resonance
Source: Membranes (Basel). 2024 May 1;14(5):105. doi: 10.3390/membranes14050105 (PMC11123086; doi:10.3390/membranes14050105)
Supplement: Supplementary file 1 [file membranes-14-00105-s001.zip › membranes-2953175-supplementary.pdf]

membranes

Supporting Information

Phospholipid Membrane Interactions of Model Ac-WL-X-LL-OH Peptides Investigated by Solid-State Nuclear Magnetic Resonance

Nicolai Etwin Alsaker [1,*], Øyvind Halskau [2], Bengt Erik Haug [1], Nathalie Reuter [1,3] and Willy Nerdal [1]

[1] Department of Chemistry, University of Bergen, Allégaten 41, N-5007 Bergen, Norway; bengt-erik.haug@uib.no (B.E.H.); nathalie.reuter@uib.no (N.R.); willy.nerdal@uib.no (W.N.)
[2] Department of Biological Sciences, University of Bergen, Thormøhlens gate 53A, N-5006 Bergen, Norway; oyvind.halskau@uib.no
[3] Computational Biology Unit, Department of Informatics, University of Bergen, Thormøhlens gate 55, N-5008 Bergen, Norway
* Correspondence: nicolai.alsaker@uib.no; Tel.: +47-98624464

1. General

1.1. Chromatography

Semi-preparative high-performance liquid chromatography (HPLC) was performed on a Gilson 321 multisolvent pump (Middleton, WI, USA) with a Dionex Ultimate 3000 variable wavelength detector (Sunnyvale, CA, USA) using Waters XBridge C18 19 × 250 mm 5 µm Prep column (Paris, France) with mixtures of acetonitrile and deionized water (both containing 0.1% formic acid) as eluent at 0.55 mL/min flow. The following gradient method was used: 1–30% ACN over 3 min; 30–90% ACN over 27 min; held at 90% ACN for 3 min.

Analytical HPLC was performed on an Agilent 1290 Infinity II Flexible pump (Santa Clara, CA, USA) with a 1260 Infinity II DAD WR detector (Santa Clara, CA, USA) using a Zorbax Eclipse Plus C18 2.1 × 50mm 1.8 µm UHPLC column (Santa Clara, CA, USA) with mixtures of acetonitrile and deionized water (both containing 0.1% TFA) as eluent at 15 mL/min flow. The following gradient method was used: 1–30% ACN over 1 min; 30–90% ACN over 7 min; held at 90% ACN for 1 min; return to 1% ACN over 1 min.

1.2. Mass Spectroscopy

Positive and negative ion electrospray ionization mass spectrometry was conducted on a JEOL AccuTOF™ JMS-T100LC mass spectrometer (Tokyo, Japan) that was operated with an orthogonal electrospray ionization source (ESI).

1.3. Solution NMR

All liquid NMR spectra were recorded on an 850 MHz AVANCE III HD (Fällanden, Switzerland) equipped with a 5 mm TCI CryoProbe or a 600 MHz AVANCE NEO equipped with a 5 mm QCI CryoProbe (Fällanden, Switzerland). For identification of amino acid sequences, the following spectra were acquired (Bruker experiment title): 1D ^{1}H (*zgesgppe*), COSY (*cosygpppqf*), DIPSI (*dipsi2eqgpph*) or TOCSY (*mlevphpp*), HSQC (*hsqcedetgpsisp2.3*), HMBC (*hmbcgpl3nd*), ROESY (*roesyphpp.2*) or NOESY (*noesygpphpp*) and ^{15}N HSQC (*hsqcetf3gpsi*). All chemical shifts (δ) are given in ppm and referenced to residual CD_3CN or $(CD_3)_2SO$ solvent signal. Spectra were processed using Bruker TopSpin® 4.1.1, MestreNova® 14.2.0 and Sparky.[1]

1.4. Peptide Synthesis

Manual peptide synthesis. H-Leu-HMPB-ChemMatrix® resin (Biotage, Uppsala, Sweden; 35–100 mesh, 0.33–0.51 mmol/g loading, typically 0.500 g) was swelled in DMF for

30 min. Fmoc-protected amino acid (3 equiv.) and HCTU (3 equiv.) were dissolved in DMF (approx. 1 mL) and DIPEA (6 eq.) was added. The mixture was added to the resin and additional DMF was added to cover the resin. After agitation for 20 min at rt, the reaction mixture was drained off and the resin was washed with three fresh portions of DMF (3× bed volume) with 1 min agitation. Fmoc deprotections were performed using piperidine in DMF (20% *v*/*v*, 3× bed volume) for 10 min, followed by DMF washing. All peptides were acetylated using a mixture of Ac_2O/DIPEA/DMF (10:5:85 *v*/*v*, 3× bed volume) with agitating for 1 h followed by DMF washing. The resin was washed further with three portions of CH_2Cl_2 (3× bed volume), three portions of MeOH (3× bed volume) and three portions of Et_2O (3× bed volume) and dried under reduced pressure for 30 min.

Microwave-assisted peptide synthesis was carried out on a Biotage® Initiator+ Alstra automated microwave peptide synthesizer (Uppsala, Sweden). H-Leu-HMPB-ChemMatrix® resin (Biotage, Uppsala, Sweden; 35-100 mesh, 0.33–0.51 mmol/g loading, typically 0.500 g) was swelled in DMF (9 mL) at 70 °C for 20 min in a 30 mL Biotage® reactor (Uppsala, Sweden). Coupling of amino acids and Fmoc-deprotection were performed as described above using amino acid concentrations of 0.2 M in DMF at 75 °C for 15 min for couplings and at rt for 10 min for Fmoc-deprotection. Acetylation and final washing were performed manually as described above.

Cleavage and purification. The resin was treated with TFA/TIS/H_2O (95:2.5:2.5 *v*/*v*, 3× bed volume) under gentle agitation for at least 3 h. Next, the resin was filtered off and washed three times with fresh TFA (3× bed volume), and the combined filtrates were concentrated under reduced pressure. Cold Et_2O was added to precipitate the crude product, which was triturated with fresh cold Et_2O three times, dried under reduced pressure and purified by semi-preparative RP-HPLC to at least 95% purity as confirmed by HPLC (220 nm). The molecular composition was confirmed by HRMS and the structure and amino acid sequence were confirmed by solution NMR.

2. Peptide Synthesis Analytical Data

2.1. Ac-WL-L-LL-OH:

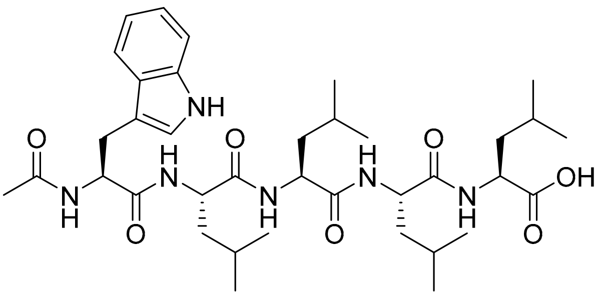

HRMS (ESI+): *m*/*z* $[M+Na]^+$ calcd. for $C_{37}H_{59}N_6O_7Na^+$ 721.4259; Found 721.4259.
t_R = 4.65 min. Purity = 95.5% (HPLC, 220 nm).

Table S1. Ac-WL-L-LL-OH ^1H resonance table in $(CD_3)_2SO$.

Residue	NH	H^{α}	H^{β}	H^{γ}	H^{δ}	Other
Ac						CH_3 1.75
W	8.02	4.52	3.07, 2.88		7.12	$NH^{\varepsilon1}$ 10.77; $H^{\varepsilon3}$ 7.61; $H^{\zeta2}$ 7.31; $H^{\zeta3}$ 6.96; H^{η} 7.05
L	8.08	4.29	1.453	1.563	0.875; 0,83	
L	7.89	4.31	1.444	1.592	0.871; 0,83	
L	7.83	4.32	1.435	1.591	0.867; 0.82	
L	7.94	4.18	1.53, 1.48	1.614	0.873; 0.82	CO_2H 12.48

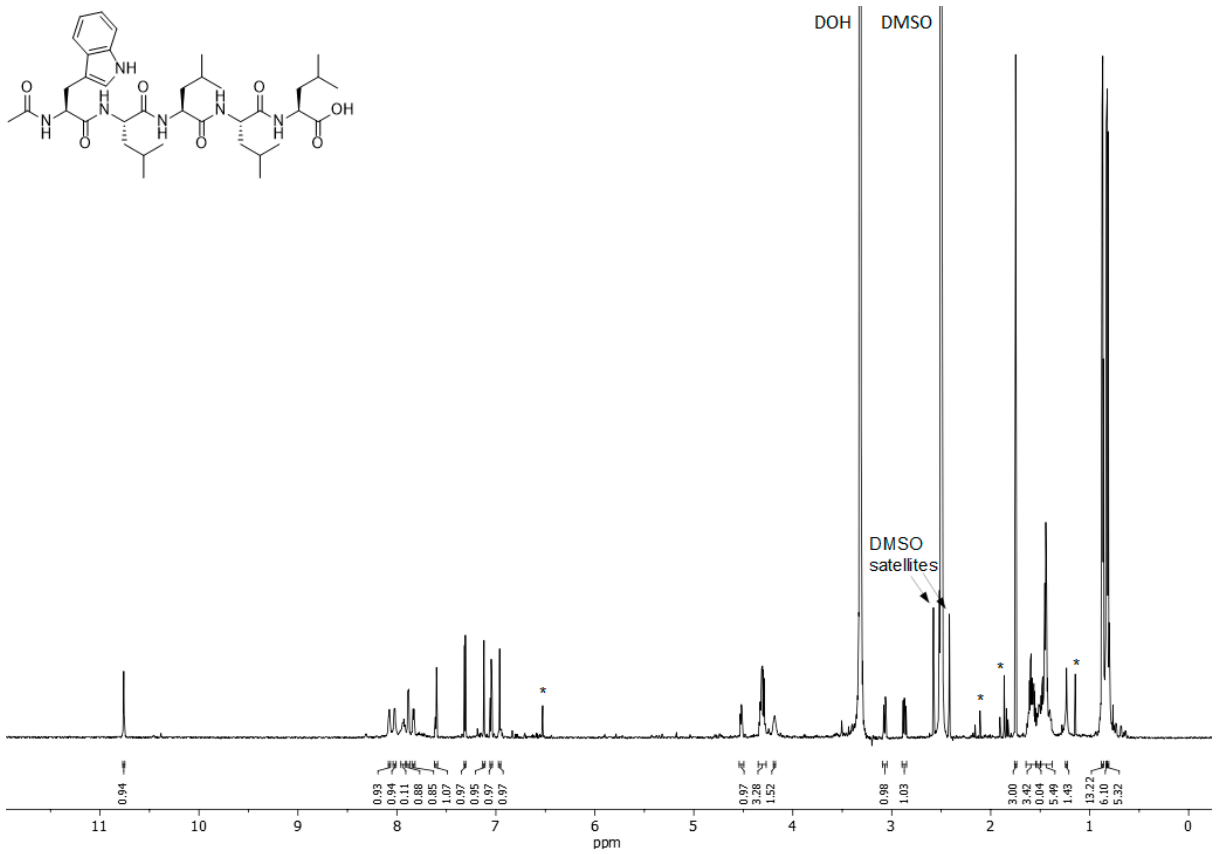

Figure S1. ^{1}H NMR spectrum of Ac-WL-L-LL-OH in $(CD_3)_2SO$. Minor impurities are marked.

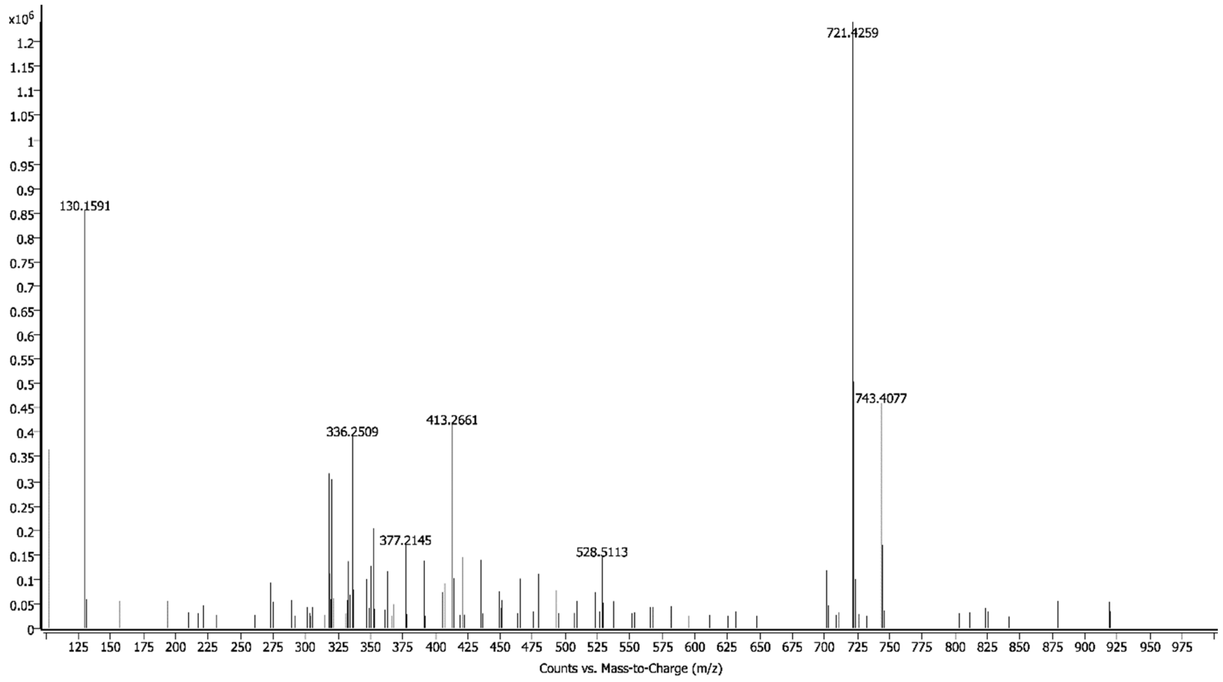

Figure S2. HRMS ESI+ spectrum of Ac-WL-L-LL-OH.

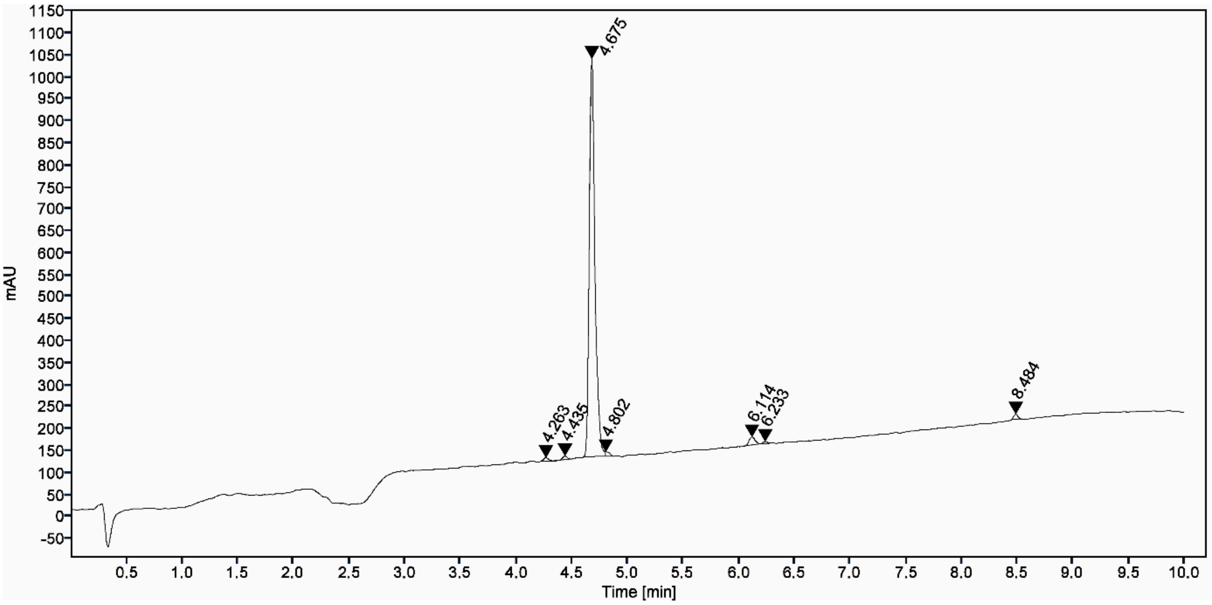

Figure S3. Chromatogram (220 nm) of Ac-WL-L-LL-OH.

2.2. Ac-WL-Y-LL-OH:

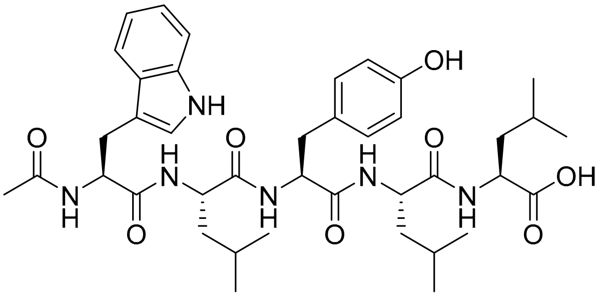

HRMS (ESI+): *m/z* $[M+Na]^+$ calcd. for $C_{40}H_{56}N_6O_8Na^+$ 771.4057; Found 771.4050.
t_R = 3.96 min. Purity = 99.2% (HPLC, 220 nm).

Table S2. Ac-WL-Y-LL-OH ^1H resonance table in CD_3CN.

Residue	NH	H^{α}	H^{β}	H^{γ}	H^{δ}	Other
Ac						CH_3 1.86
W	7.23	4.57	3.20, 3.04		7.10	$NH^{\varepsilon 1}$ 9.65; $H^{\varepsilon 3}$ 7.56; $H^{\zeta 2}$ 7.36; $H^{\zeta 3}$ 7.10; H^{η} 7.02
L	7.42	4.16	1.38, 1.32	1.24	0.777; 0.73	
Y	7.57	4.34	2.99, 2.79		7.02	$H^{\varepsilon 1}$ 6.70; OH^{η} -
L	7.45	4.24	1.51, 1.41	1.17	0.784; 0.72	
L	7.55	4.30	1.68, 1.57	1.67	0.88; 0.83	CO_2H -

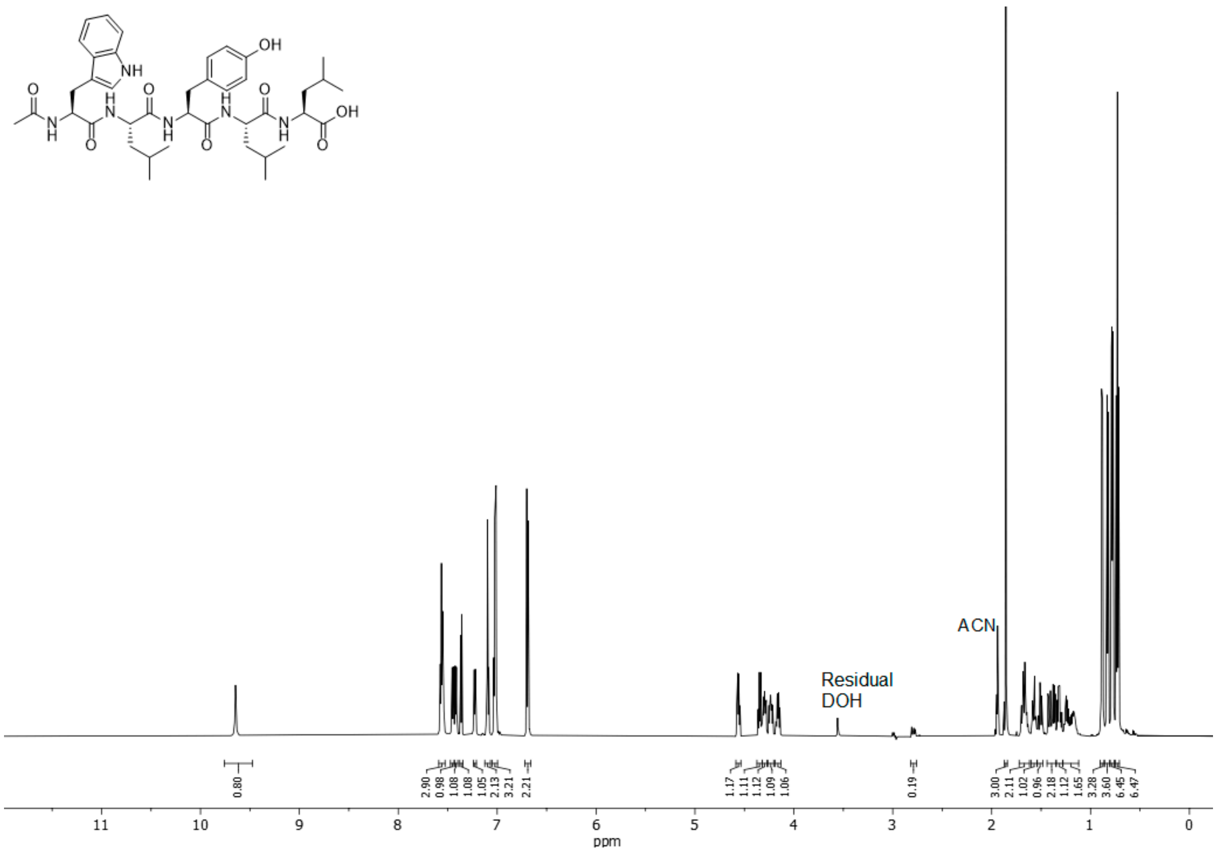

Figure S4. ^{1}H-NMR spectrum of Ac-WL-Y-LL-OH in CD_3CN. Water suppression also suppresses H$^{\beta}$ signals of the aromatic residues.

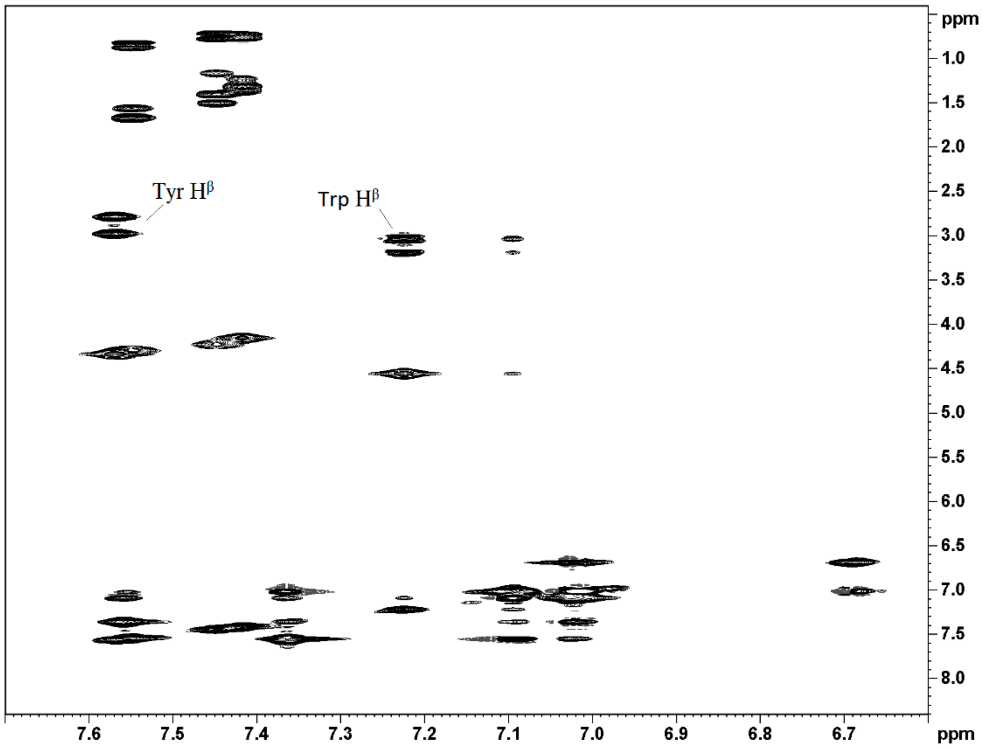

Figure S5. TOCSY spectrum of the NH region in F2 shows aromatic H$^{\beta}$ suppressed during 1D ^{1}HNMR with water suppression of Ac-WL-Y-LL-OH in CD_3CN.

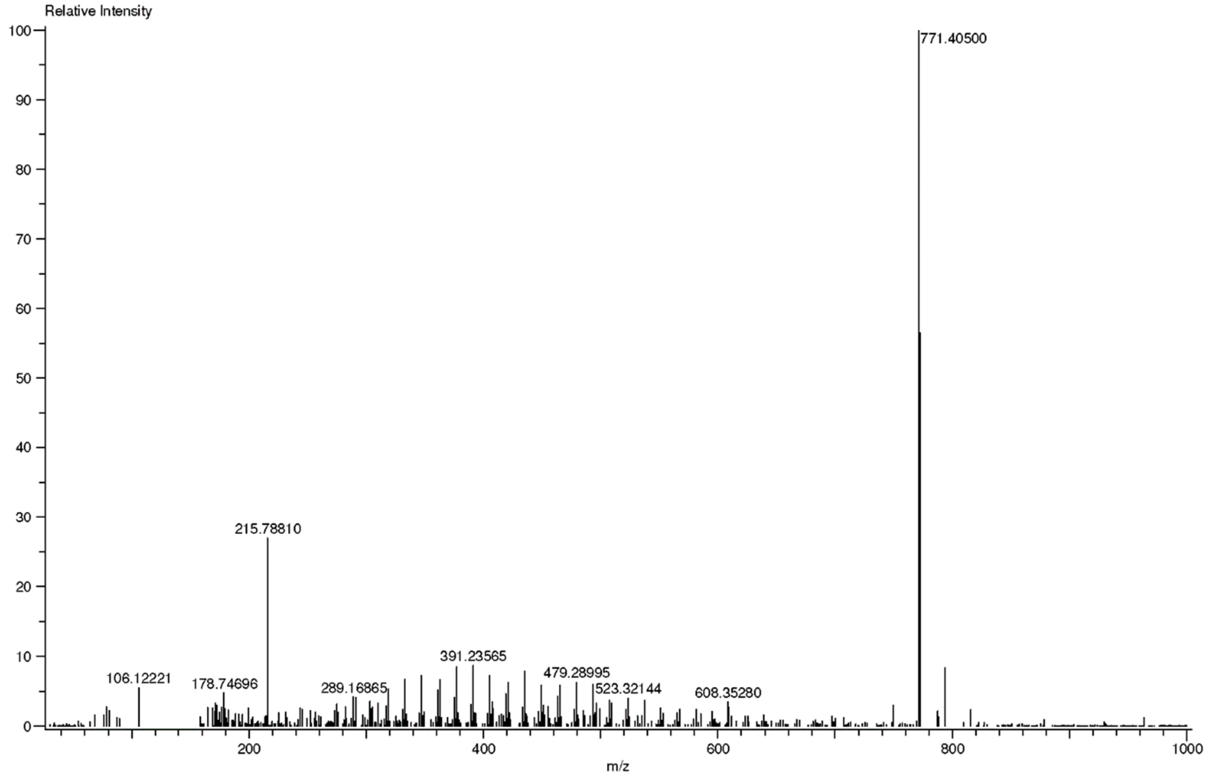

Figure S6. HRMS ESI+ spectrum of Ac-WL-Y-LL-OH.

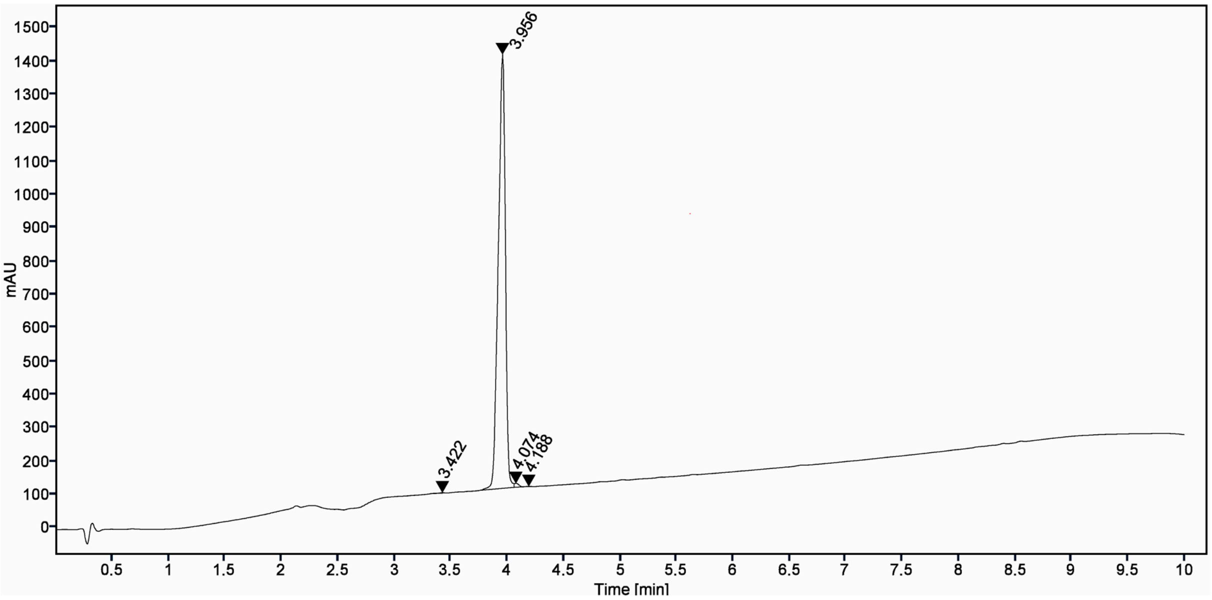

Figure S7. Chromatogram (220 nm) of Ac-WL-Y-LL-OH.

2.3. Ac-WL-F-LL-OH:

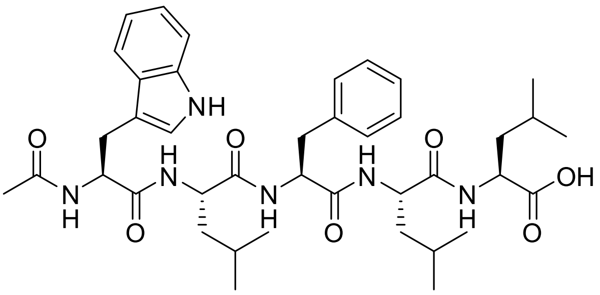

HRMS (ESI+): *m/z* $[M+Na]^+$ calcd. for $C_{40}H_{56}N_6O_7Na^+$ 755.4103; Found 755.4104.
t_R = 4.82 min. Purity = 95.5% (HPLC, 220 nm).

Table S3. Ac-WL-F-LL-OH ^{1}H resonance table in $(CD_3)_2SO$.

Residue	NH	H$^{\alpha}$	H$^{\beta}$	H$^{\gamma}$	H$^{\delta}$	Other
Ac						CH_3 1.75
W	8.03	4.50	3.05, 2.88		7.10	NH$^{\varepsilon1}$ 10.73; H$^{\varepsilon3}$ 7.59; H$^{\zeta2}$ 7.31; H$^{\zeta3}$ 7.04; H$^{\eta}$ 6.96
L	8.02	4.23	1.21	1.28	0.76; 0.73	
F	8.16	4.55	2.99, 2.75		7.24	H$^{\varepsilon}$ 7.21; H$^{\zeta}$ 7.16
L	8.14	4.33	1.44	1.46	0.82; 0.78	
L	8.08	4.16	1.55, 1.49	1.65	0.87; 0.81	CO_2H 12.49

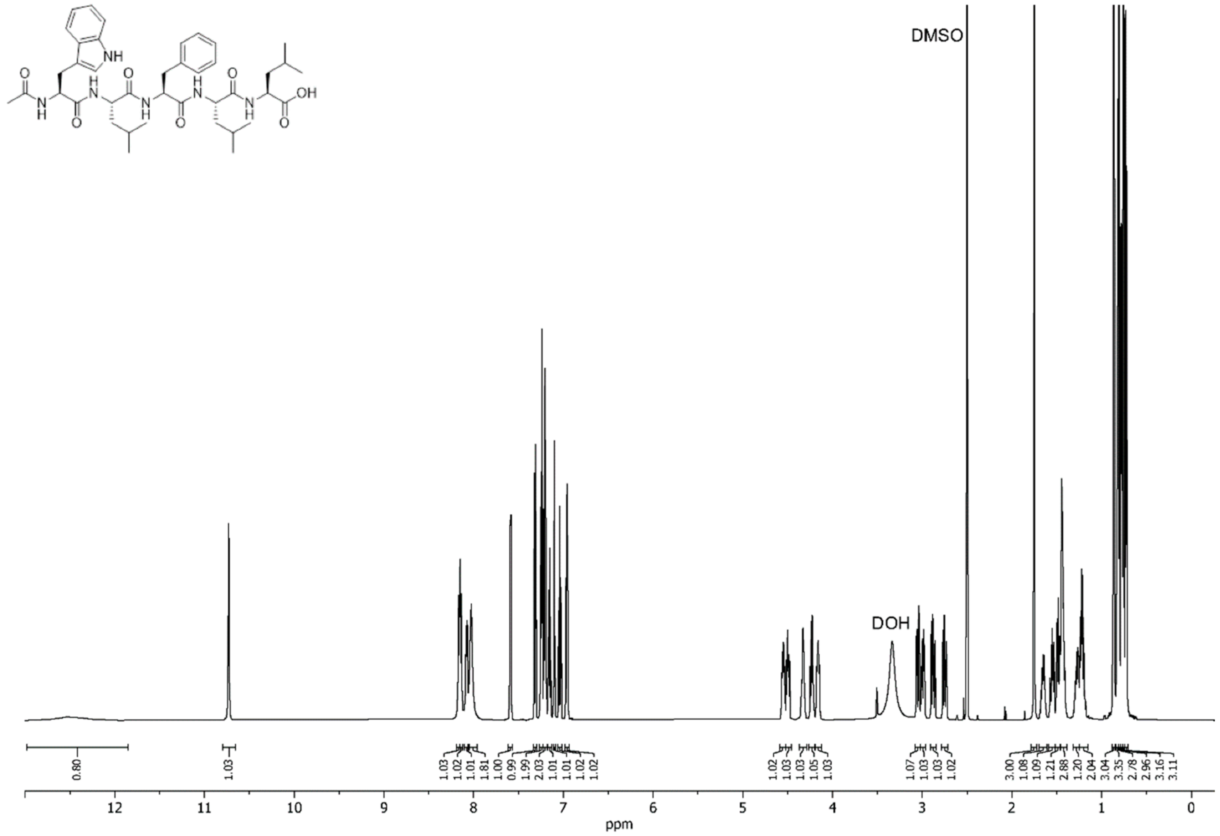

Figure S8. ^{1}H NMR spectrum of Ac-WL-F-LL-OH in $(CD_3)_2SO$.

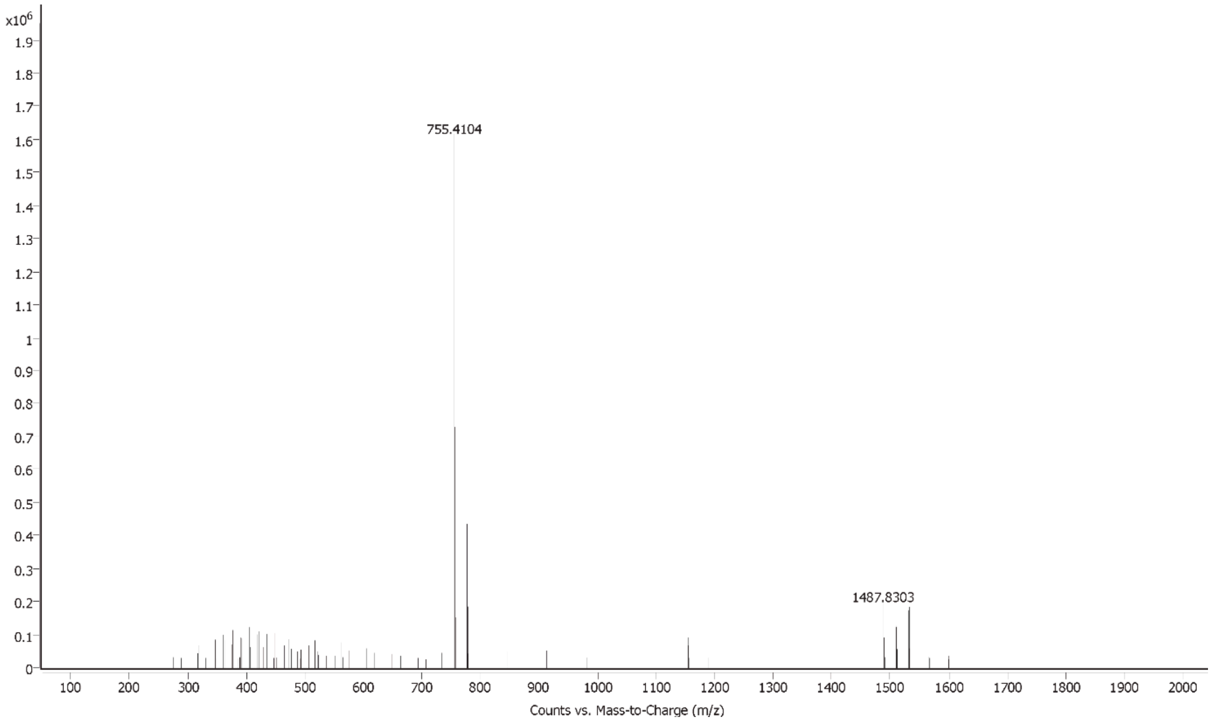

Figure S9. HRMS ESI+ spectrum of Ac-WL-F-LL-OH.

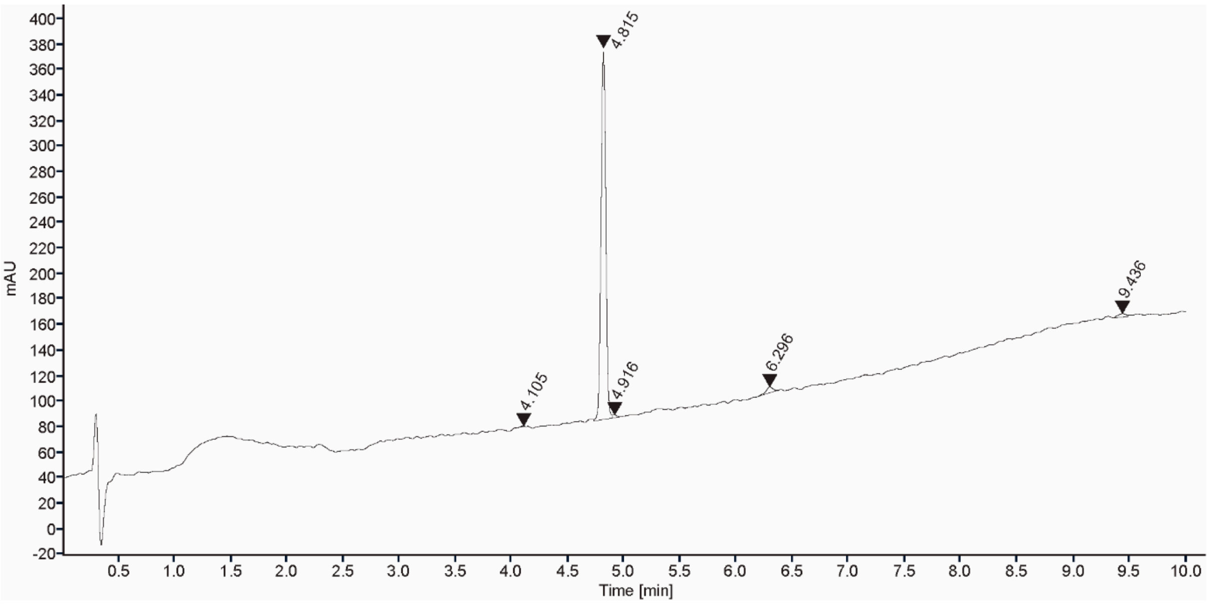

Figure S10. Chromatogram (220 nm) of Ac-WL-F-LL-OH.

2.4. *Ac-WL-W-LL-OH:*

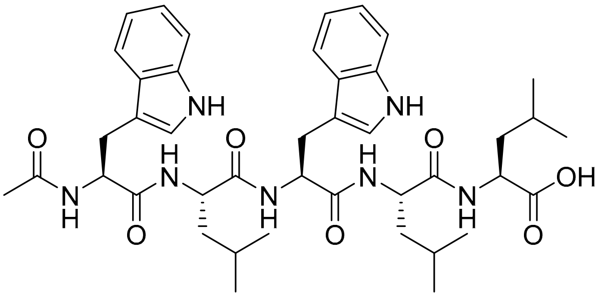

HMRS (ESI-): *m/z* [M-H]$^-$ Calcd for $C_{42}H_{56}N_7O_7^-$ 770.4246; Found 770.4247.
t_R = 4.95 min. Purity 98.2% (HPLC, 220 nm).

Table S4. Ac-WL-W-LL-OH ^{1}H resonance table in $(CD_3)_2SO$.

Residue	NH	H^α	H^β	H^γ	H^δ	Other
Ac						CH_3 1.75
W	8.00	4.51	3,07, 2.87		7.11	NH^{ε1} 10.73; H^{ε3} 7.60; H^{ζ2} 7.30; H^{ζ3} 6.95; H^η 7.04
L	8.02	4.26	1.38	1.52	0.84; 0.79	
W	7.94	4.57	3.14, 2.96		7.11	NH^{ε1} 10.75; H^{ε3} 7.55; H^{ζ2} 7.30; H^{ζ3} 6.95; H^η 7.04
L	7.93	4.37	1.45	1.59	0.86; 0.81	
L	7.97	4.21	1.53, 1.49	1.64	0.88; 0.83	CO_2H 12.47

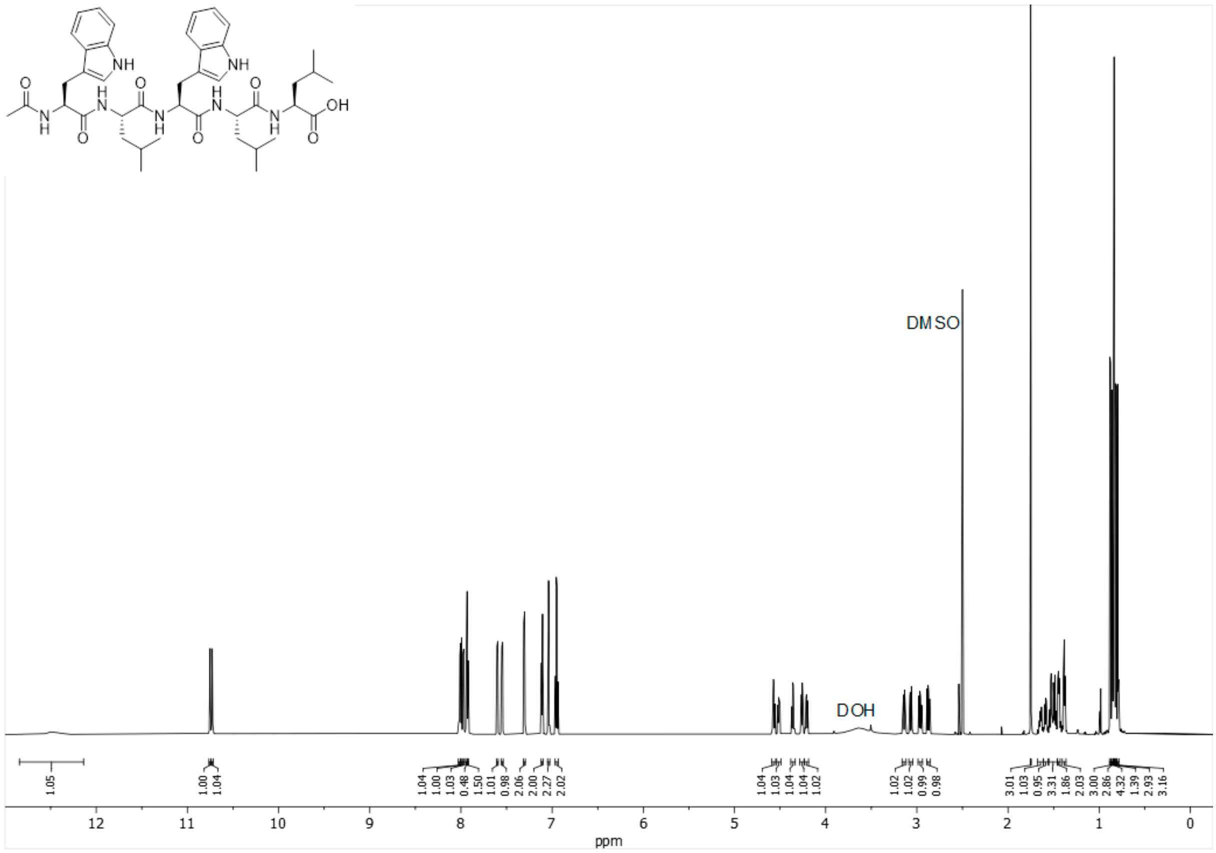

Figure S11. ^{1}H NMR spectrum of Ac-WL-W-LL-OH in $(CD_3)_2SO$.

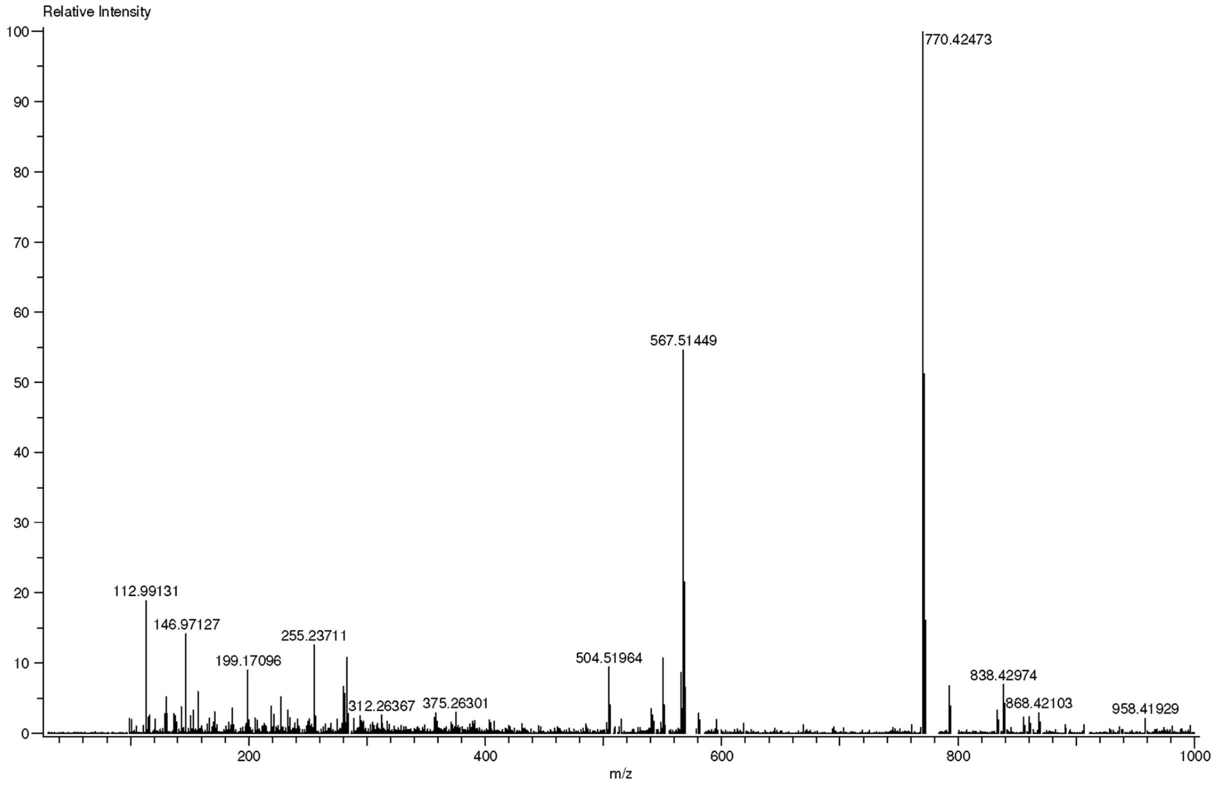

Figure S12. HRMS ESI- spectrum of Ac-WL-W-LL-OH.

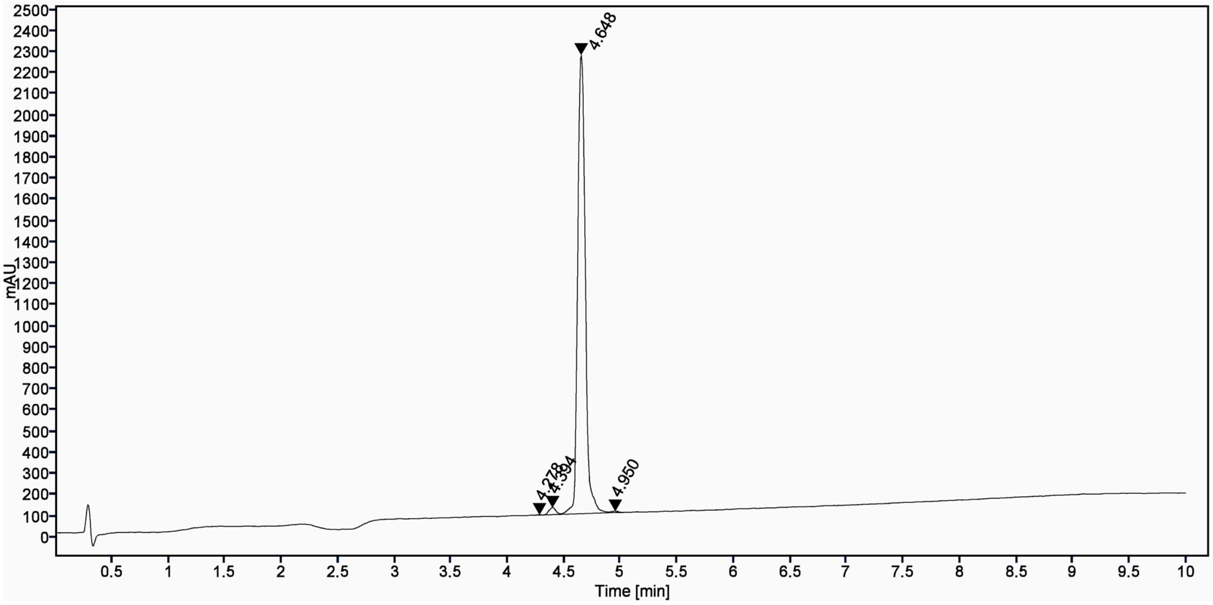

Figure S13. Chromatogram (220 nm) of Ac-WL-W-LL-OH.

3. Solid-State NMR Spectral Data

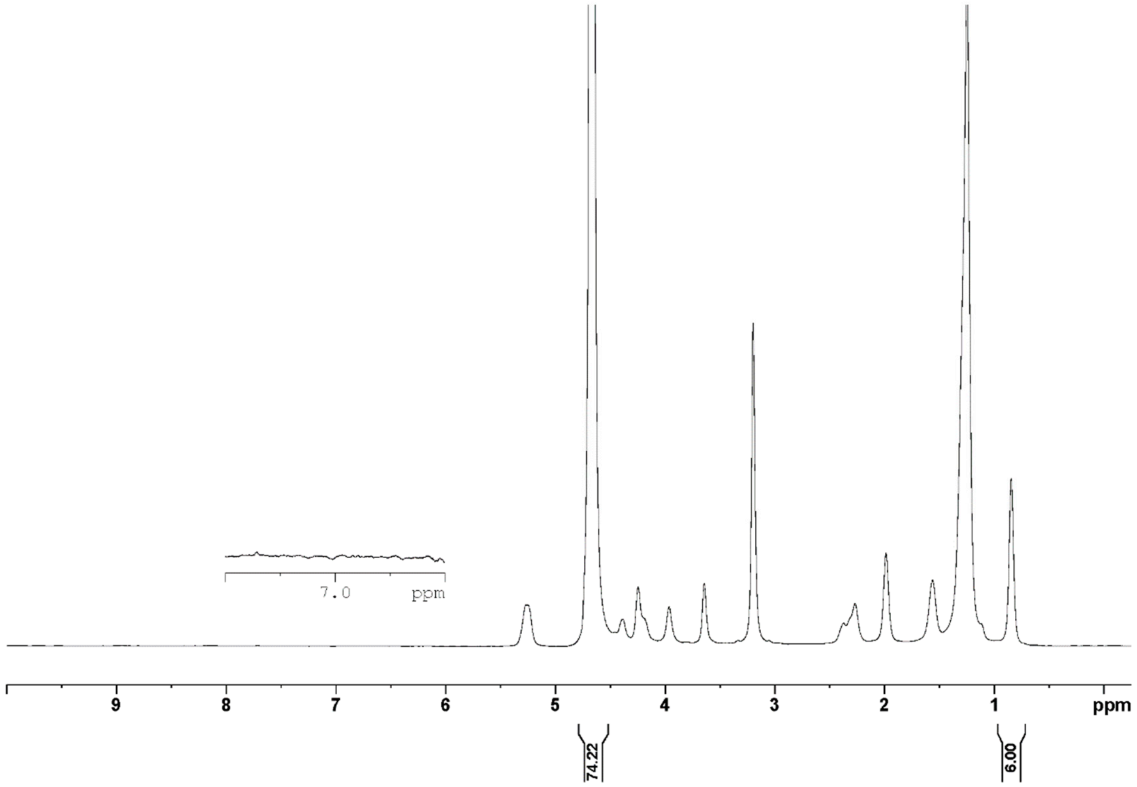

Figure S14. Pure POPC ¹H NMR MAS (6 kHz) spectrum. Sample hydration was determined from the water peak and lipid methyl-termini integrals. Processed with 3 Hz line broadening.

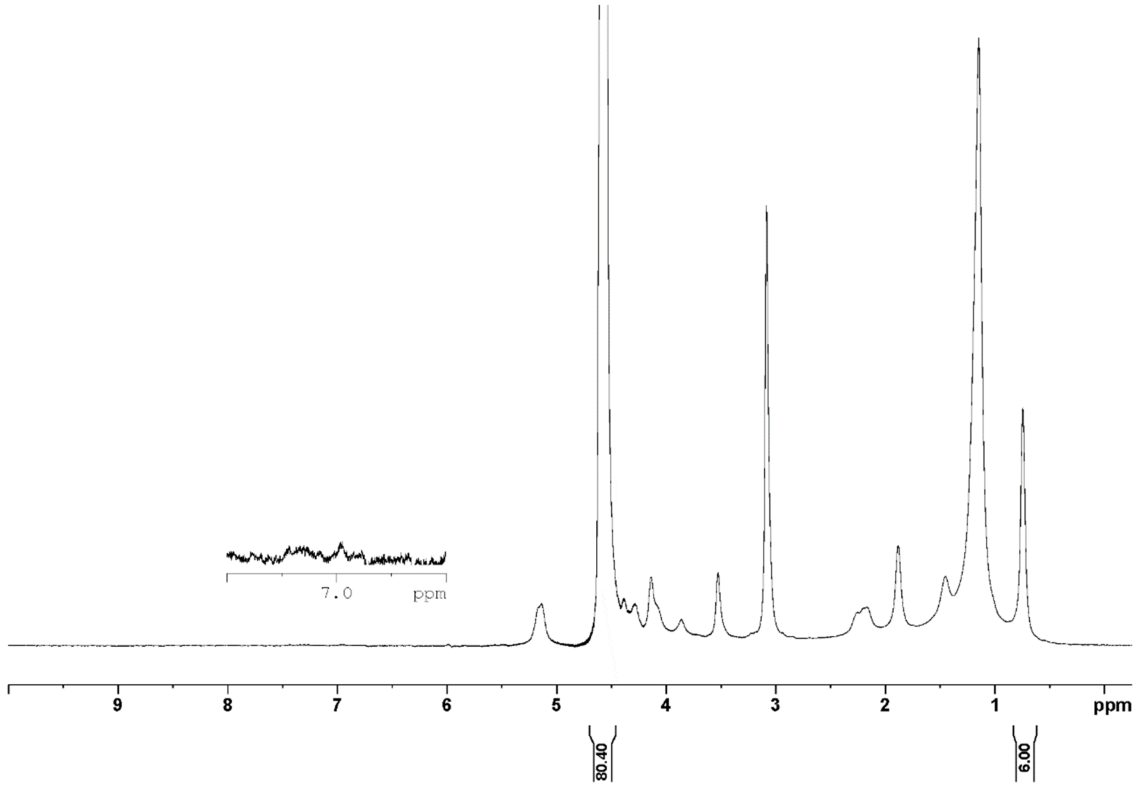

Figure S15. ¹H NMR MAS spectrum hydrated POPC sample doped with Ac-WL-L-LL-OH (10 mol %). Residual peptide signals (Ar-H, NH) are shown in the 6-8 ppm window.

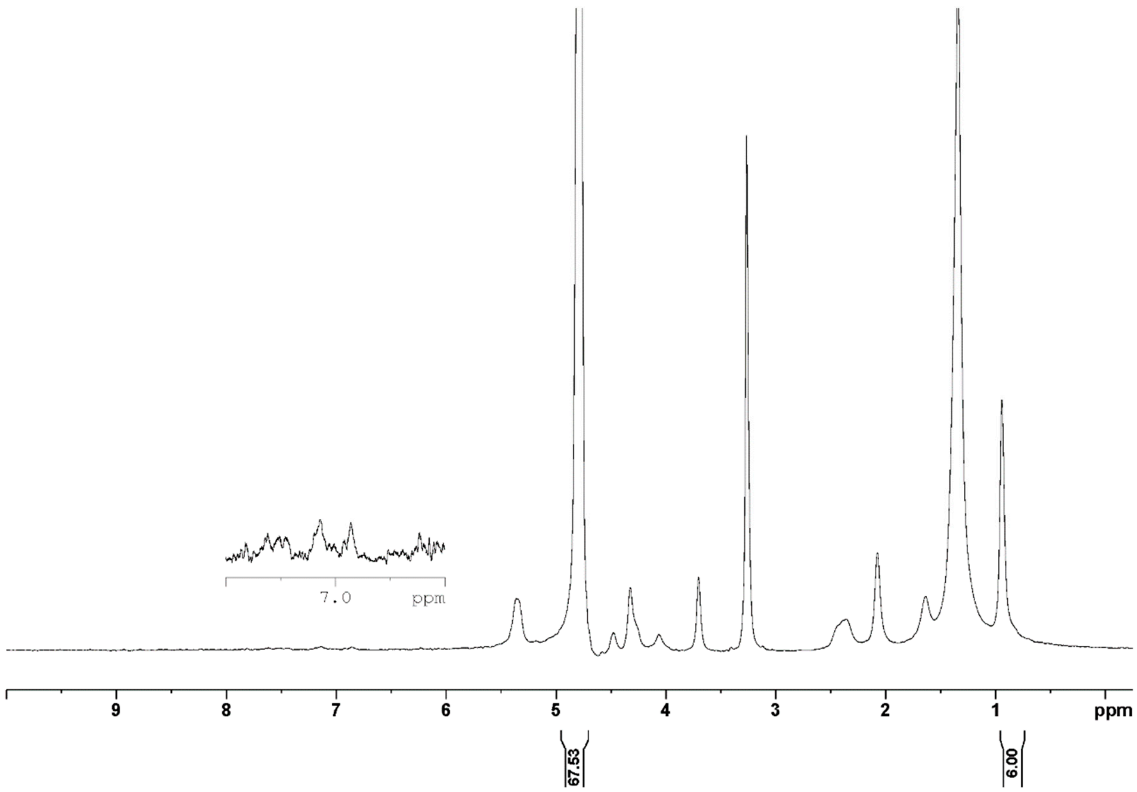

Figure S16. ^{1}H NMR MAS spectrum hydrated POPC sample doped with Ac-WL-Y-LL-OH (10 mol %). Residual peptide signals (Ar-H, NH) are shown in the 6-8 ppm window.

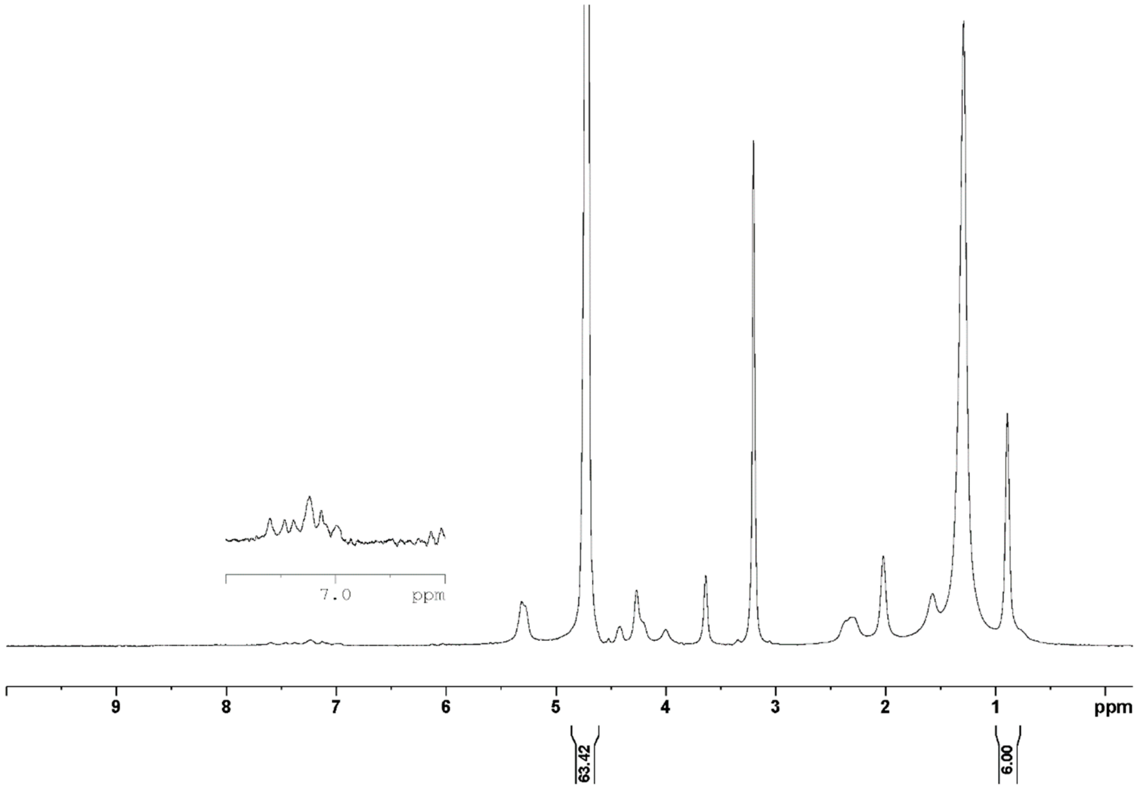

Figure S17. ^{1}H NMR MAS spectrum hydrated POPC sample doped with Ac-WL-F-LL-OH (10 mol %). Residual peptide signals (Ar-H, NH) are shown in the 6-8 ppm window.

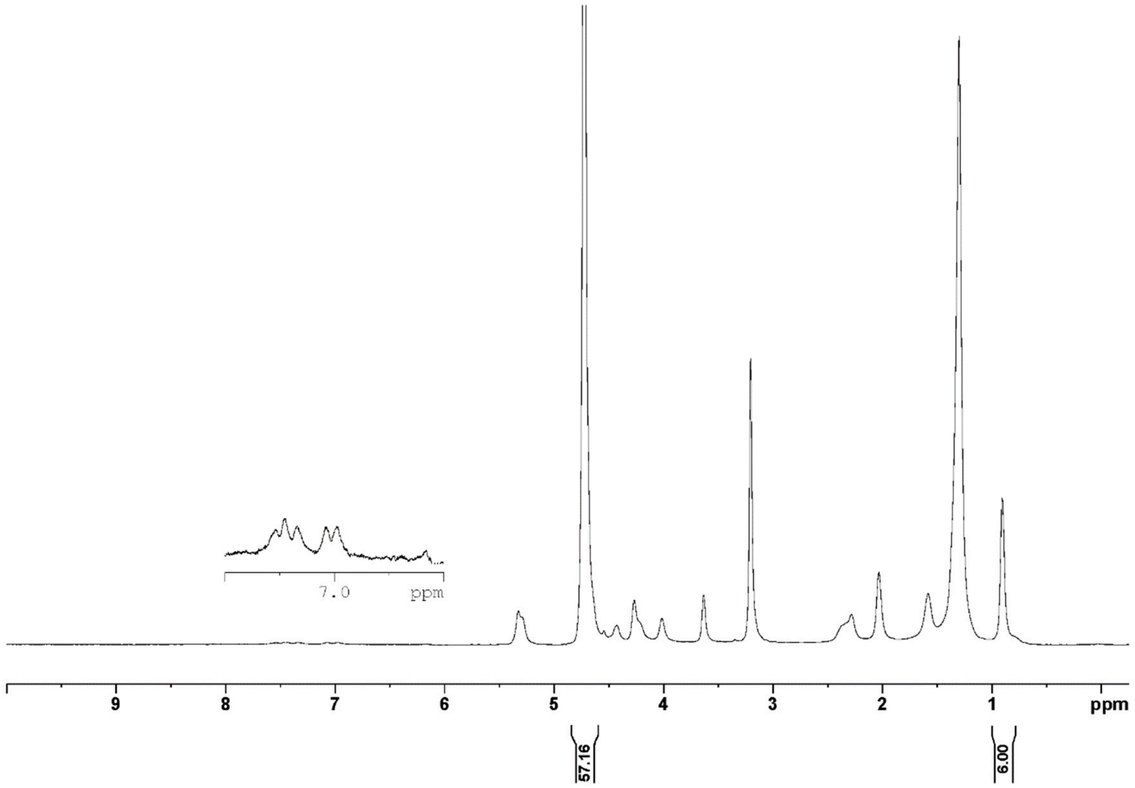

Figure S18. ^{1}H NMR MAS spectrum hydrated POPC sample doped with Ac-WL-W-LL-OH (10 mol %). Residual peptide signals (Ar-H, NH) are shown in the 6-8 ppm window.

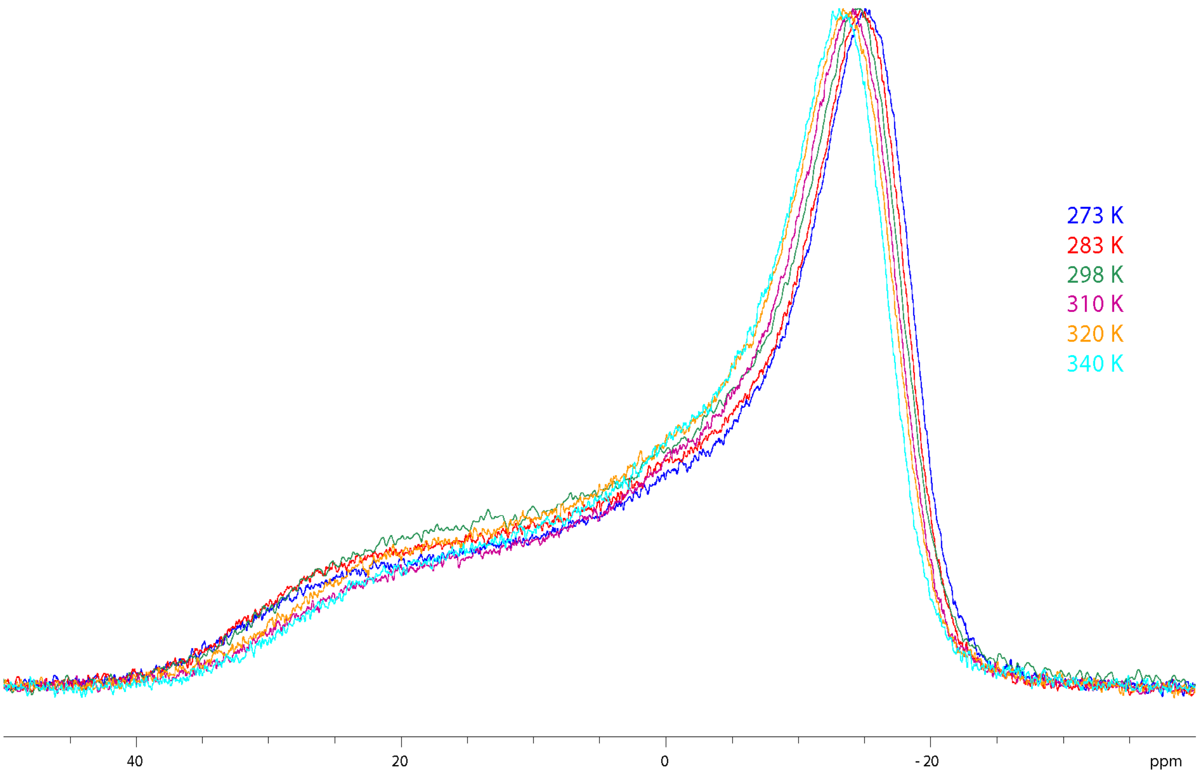

Figure S19. Static ^{31}P NMR line-shape of pure POPC reference sample at select temperatures without BB decoupling.

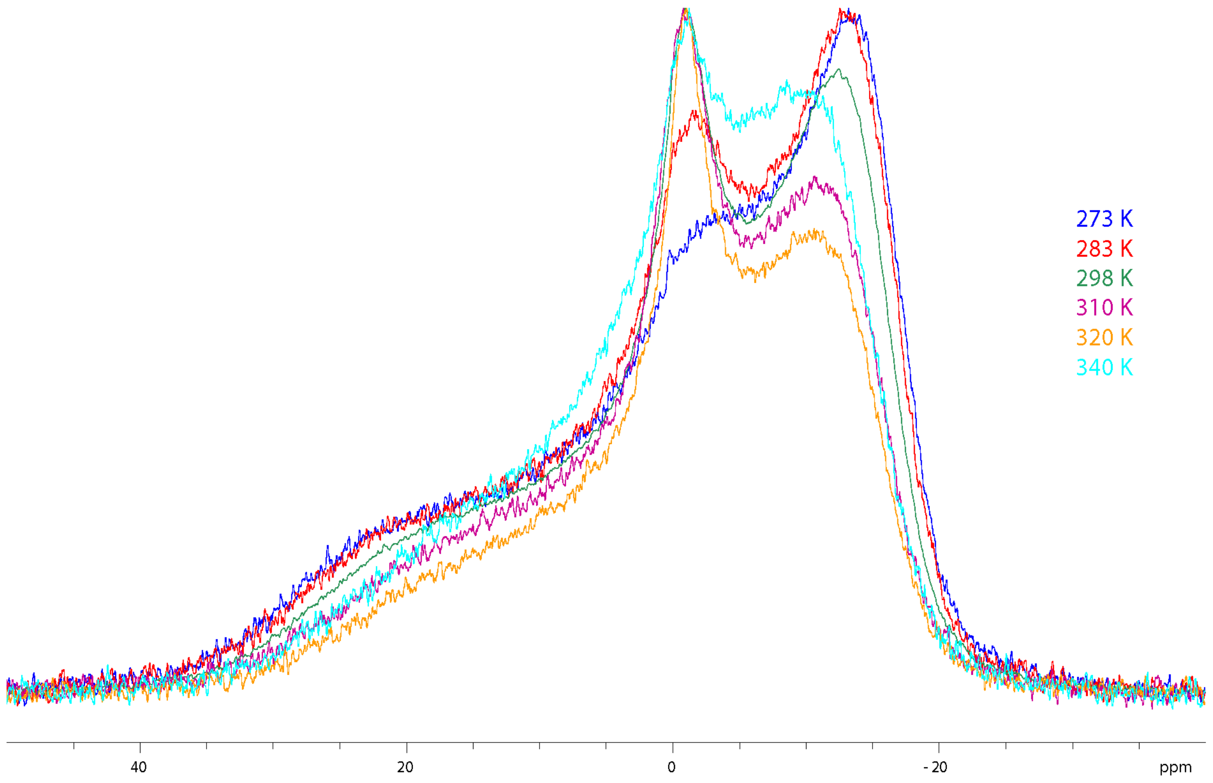

Figure S20. Static ^{31}P NMR signal line-shape of hydrated POPC sample doped with Ac-WL-L-LL-OH (10 mol %) at select temperatures without BB decoupling.

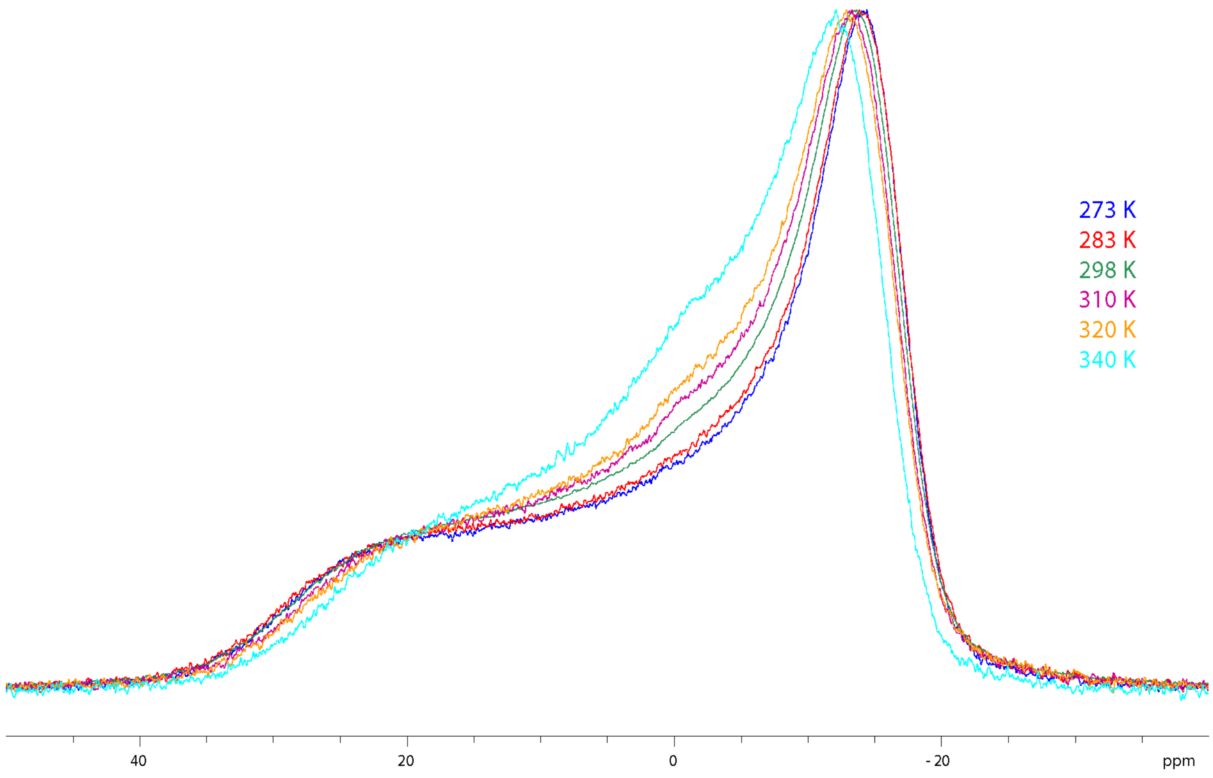

Figure S21. Static ^{31}P NMR signal line-shape of hydrated POPC sample doped with Ac-WL-Y-LL-OH (10 mol %) at select temperatures without BB decoupling.

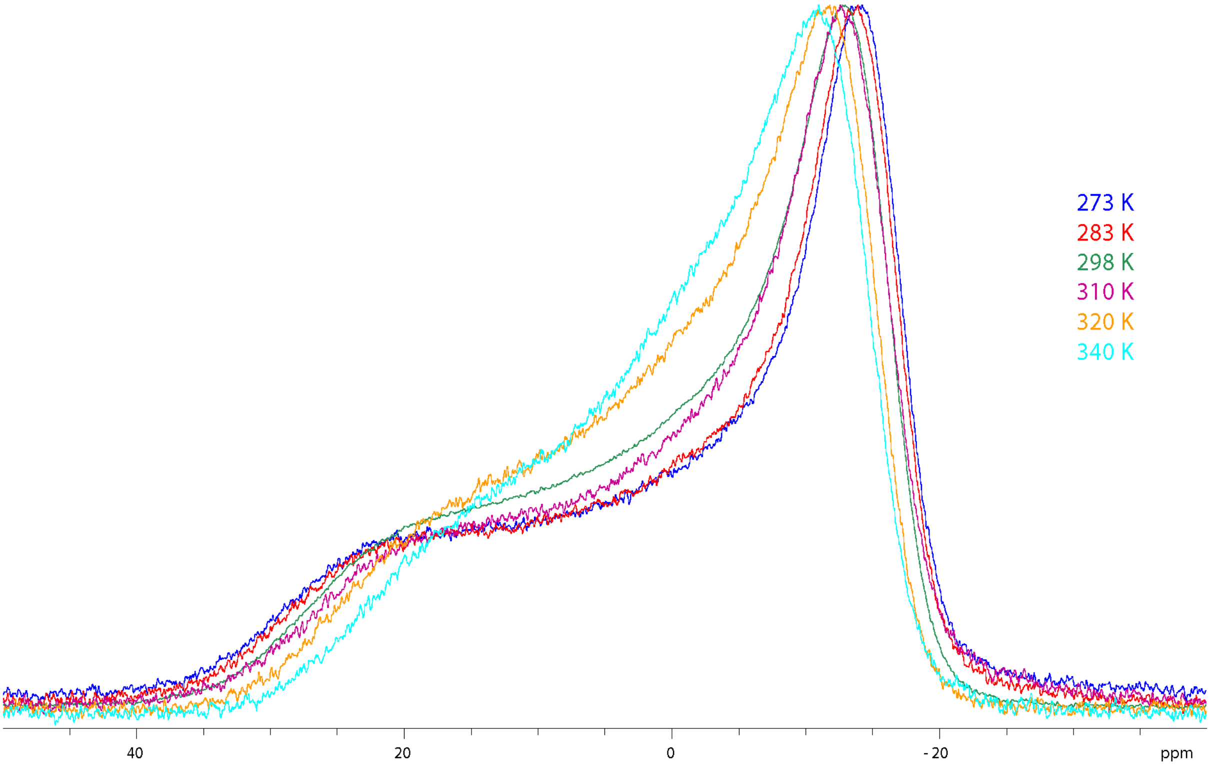

Figure S22. Static ^{31}P NMR signal line-shape of hydrated POPC sample doped with Ac-WL-F-LL-OH (10 mol %) at select temperatures without BB decoupling.

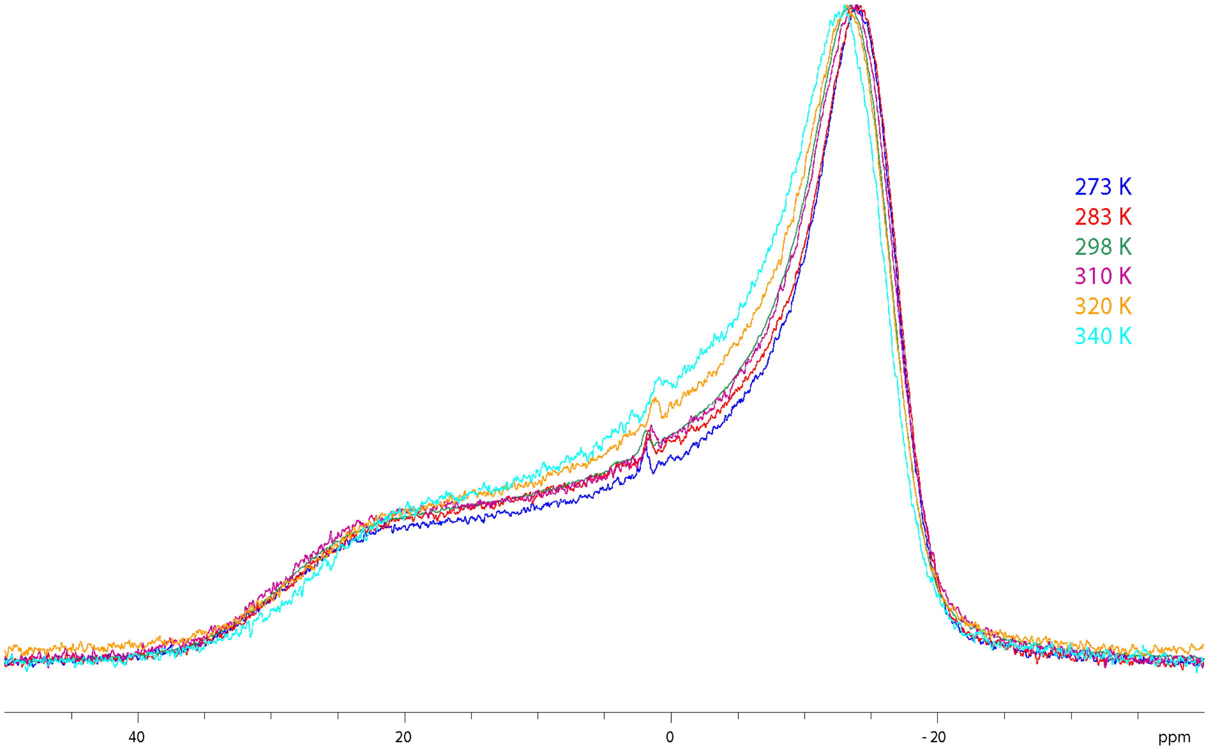

Figure S23. Static ^{31}P NMR signal line-shape of hydrated POPC sample doped with Ac-WL-W-LL-OH (10 mol %) at select temperatures without BB decoupling.

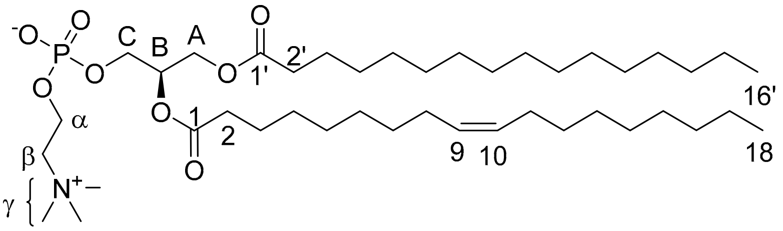

Figure S24. POPC carbon labelling.

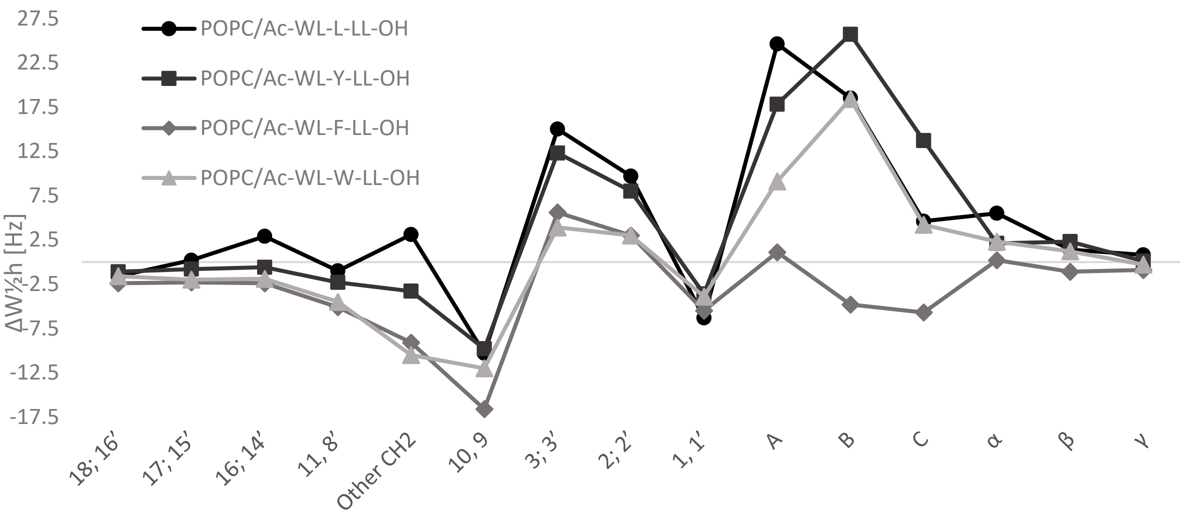

Figure S25. Peak width at half-height of specific ^{13}C MAS signals originating from hydrated POPC samples doped with Ac-WL-X-LL-OH (10 mol%) relative to pure POPC.

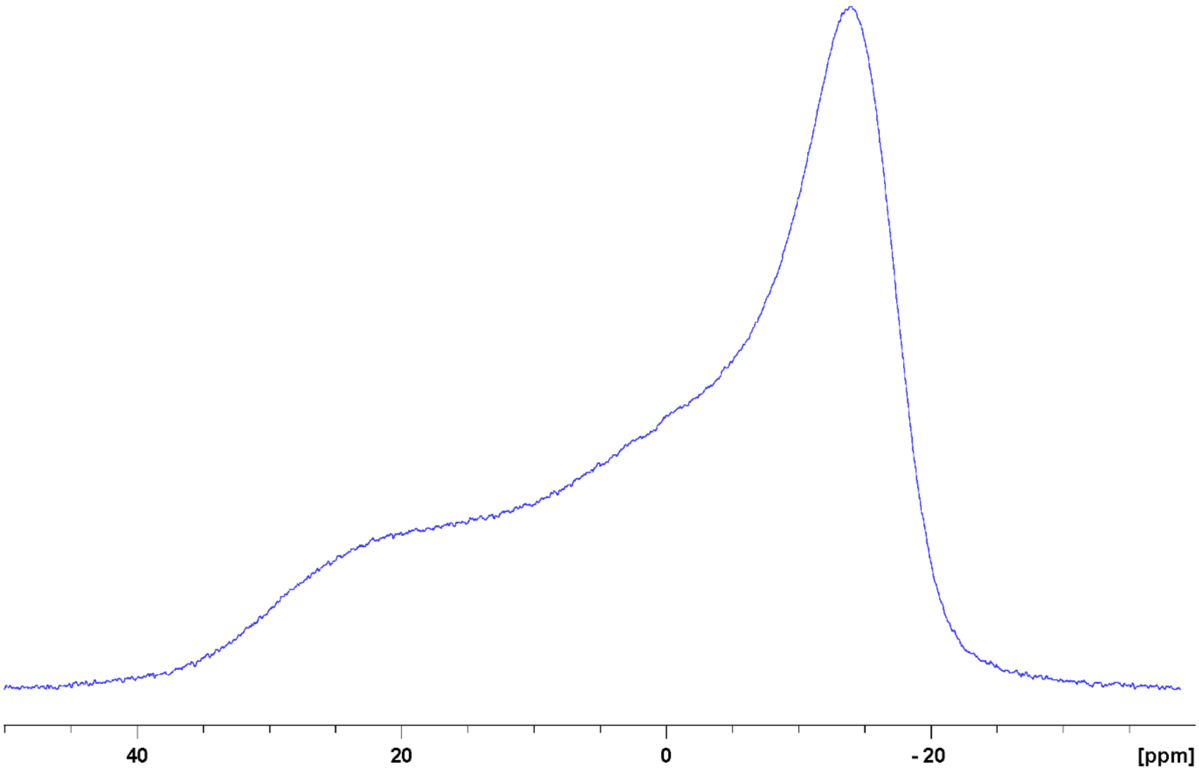

Figure S26. Static ^{31}P NMR signal line-shape of hydrated POPC sample doped with Ac-WL-L-LL-NH_2 (10 mol %) at 298 K without BB decoupling.

References

1. Lee, W.; Tonelli, M.; Markley, J. L., NMRFAM-SPARKY: enhanced software for biomolecular NMR spectroscopy. *Bioinformatics* **2014,** *31*, 1325–1327.
